# Psychiatric Symptom Profiles Predict Functional Impairment

**DOI:** 10.3389/fpsyt.2019.00037

**Published:** 2019-02-13

**Authors:** Joachim Tanner, Thomas Zeffiro, Daniela Wyss, Noelle Perron, Michel Rufer, Christoph Mueller-Pfeiffer

**Affiliations:** ^1^Department of Consultation-Liaison-Psychiatry and Psychosomatic Medicine, University Hospital Zurich, University of Zurich, Zurich, Switzerland; ^2^Neurometrika, Potomac, MD, United States; ^3^Department of Diagnostic Radiology and Nuclear Medicine, University of Maryland, Baltimore, MD, United States; ^4^Center of Education and Research (COEUR), Psychiatric Services of the County of St. Gallen-North, Wil, Switzerland; ^5^Clinical Psychology and Psychotherapy, University of Bern, Bern, Switzerland; ^6^Department of Psychiatry, Psychotherapy, and Psychosomatics, Psychiatric Hospital Zurich, University of Zurich, Zurich, Switzerland

**Keywords:** disability, functional impairment, insurance medicine, legal assessment, daily functioning, occupational health

## Abstract

**Objective:** Mental illness often interferes with daily functioning and an individual's pattern of psychiatric signs and symptoms may predict risk of future disability. Understanding the linkage between psychiatric symptoms and impaired functioning is critical for accurate rehabilitation planning and legal assessment. Here, we investigated the stability of functional impairment measures over 18 months and their association with psychiatric symptoms. Moreover, we developed a clinical self-report measure that allows estimation of functional impairment levels over 18 month observation periods.

**Methods:** Consecutively treated outpatients and daycare patients (*N* = 155) from several psychiatric units in Switzerland completed the Dissociative Experiences Scale, Somatoform Dissociation Questionnaire, Multidimensional Inventory for Dissociation, Beck Depression Inventory, Brief Symptom Inventory, and WHO Disability Assessment Schedule at baseline, 6, 12, and 18 month follow-up examinations. The association between symptoms functional impairment over time was investigated using longitudinal linear mixed models. Penalized regression was used to identify questionnaire items that best predicted functional impairment.

**Results:** We found high stability in the extent of functional impairment over 18 months. Fear of negative evaluation, fatigue, concentration problems, negative alterations in mood, and dissociative symptoms showed the strongest association with functional impairment measures. The empirically derived scale for functional impairment prediction explained between 0.62 and 0.77 of the variance in disability across various life domains.

**Conclusion:** Given the capability for somatic and mental symptoms associated with social anxiety, depression, and dissociation to predict future disability, these symptoms have strong potential for guiding rehabilitation planning and prognostic evaluation in insurance medicine. The Functional Impairment Prediction Scale may serve as a valuable, empirical-based extension in legal assessments of how work capacity is affected by psychological factors.

## Introduction

Mental illness often interferes with daily functioning and work ability. Worldwide, psychiatric disability accounts for the highest proportion of disability among all diseases (1). In Switzerland, 43% of disability payments newly granted in the last decade are associated with mental disorders (2). Data in other West European countries, America and Australia are similar, and in most countries an increasing percentage of disability payments are granted due to mental disorders. A study conducted by the Organization for Economic Cooperation and Development (OECD) in West European countries, America and Australia reported an overall increase of disability payments due to mental disorders from the mid-1990s through 2009. In 2009, the proportion of disability payments due to mental disorders were between 24.9% (Norway) and 44.2% (Denmark). In a majority of countries, this rate increased by more than a factor of two during the observation period. Only the Netherlands and the United States showed slightly decreasing rates of 0.89 and 0.95, respectively (3). Similar results has been reported by the Mental Health Economics European Network (MHEEN) (4) and in some country-specific studies. In the UK, for instance, government data from 1995 to 2014 showed a decrease of overall disability payments by 6.4%, but an increase in payments due to mental disorders from 21.4 to 45.4% (5). A cohort study conducted in the Netherlands between July 2010 and June 2011 revealed that 33% of men and 35% of women who received disability payments had a main diagnosis of a mental disorder (6). In the US, the Annual Statistical Report on the Social Security Disability Insurance of 2017 reported a rate of 35% of mental disorders among all disabled beneficiaries (7). The major challenges mental disorders represent for the health and social security systems of both developed and developing countries call for better insights into the mechanisms causing disability.

Ample evidence supports an association between mental disorders and functional impairment in various life domains. In a German cohort study, the risk for permanent disability was increased by a factor of 2.5 in individuals with depressive disorders, and by a factor of 1.3 in individuals with anxiety disorders (8). Among anxiety disorders, the highest correlations have been observed between the severity of obsessive-compulsive disorder and social anxiety disorder with levels of global disability (9). A Chinese study among patients with remitted or partially remitted major depression, residual symptoms of fatigue, psychomotor changes, sleep disturbances and weight/appetite disturbances were related to functional impairment (10). In a Spanish study of primary care patients, depressive symptoms were related to impaired social life, family life and work, anxiety was realted to impaired family life, and somatization associated with impaired functioning at work. A study among Canadian outpatients with obsessive-compulsive disorder found that obsessions, checking behavior, and hoarding had the greatest impact on daily functioning (11). Not surprisingly, psychotic symptoms contribute to impairment in individuals with schizophrenia (12, 13), with negative symptoms accounting for up to 18% of the variance in functioning (9, 14).

Mental disorders are often heterogeneous in their phenomenology. This complexity is reflected in the lists of diagnostic criteria in the Diagnostic and Statistical Manual of Mental Disorders (DSM), where some diagnoses are characterized by a considerable number of signs and symptoms. It is unlikely that all signs and symptoms respond equally well to similar treatments or contribute equally to functional impairment. For instance, the DSM-5 (15) lists 20 distinct signs and symptoms for posttraumatic stress disorder (PTSD). Of these, re-experiencing and avoidance symptoms usually improve after successful exposure therapy (16), while irritability and sleep disturbances may be more effectively treated pharmacologically (17). Almost all previous studies have investigated disability at the disorder level. Moreover, it is largely unknown which signs and symptoms clustered within a diagnosis contribute to the observed associations between mental disorders and functional impairment. There are multiple factors contributing to functional impairment, including job characteristics, psychosocial factors and functioning capacity (18). Knowing the functionally most influential signs and symptoms, would allow tailoring treatment options to optimize chances for vocational rehabilitation. Detailed knowledge concerning the influence of psychiatric signs and symptoms on work disability is also needed to achieve the most accurate assessment of individual working capability.

In psychiatric legal assessments, it is the evidence for psychopathology, not diagnosis, that provides the basis for disability payment claims (19). Basing decisions on evidence of psychopathology seems reasonable, because there is vast variability in working capacity among individuals with the same psychiatric diagnosis. In this context, medical experts must provide plausible and evidence-based explanations concerning which signs and symptoms a claimant suffers could interfere with work performance. Moreover, diagnostic criteria in classification systems such as the DSM-5 are subject to change, while sign and symptom constructs are usually better established and stable over time. Hence, evidence regarding the strength of an association between specific signs and symptoms and functional impairment is more applicable in legal assessments than is evidence relating to specific mental disorders.

The primary objective of this prospective study in outpatient and daycare patients with a broad range of psychiatric disorders was to investigate the association of functional impairment with psychiatric signs and symptoms.

## Materials and Methods

### Procedure

Participants between 18 and 65 years with sufficient fluency in the German language, who were in treatment for three or more sessions during 1/2009 to 12/2010, were eligible for study participation. Participants were recruited from two public psychiatric outpatient units, one private practice, and two psychiatric daycare units, all located in the counties of St. Gallen or Zurich in Switzerland. Exclusion criteria comprised acute psychosis, acute suicidal ideation, substance abuse with acute intoxication or withdrawal, mental retardation, and psychiatric disorders due to an underlying medical condition. The study protocol was approved by the institutional review board of the county of St. Gallen, Switzerland. All participants provided written informed consent according to the Declaration of Helsinki. Study participation was compensated.

### Measurements

Axis I and Axis II diagnoses were ascertained at baseline using the Structured Clinical Interview for DSM-IV Disorders Axis I (SCID-I) (20) and Axis II (SCID-II) (21). Dissociative disorders were ascertained using the Structured Clinical Interview for Dissociative Disorders (SCID-D-R) (22, 23). Inter-rater reliability of SCID-I and SCID-II is fair-to-excellent (24). The SCID-D-R is performed as a semi-structured interview. The categorical diagnosis of a Dissociative Disorder is based on the dimensional assessment of five dissociative symptoms “amnesia.” “depersonalization,” “derealization,” “identity confusion,” and “identity alteration” on a 4-point-Likert scale (1 = none, 2 = mild, 3 = moderate, 4 = severe). The interviewer rates the severity of each of the five dissociative symptoms according to specific behaviors and experiences reported by the patient as well as the observation of dissociative symptoms during the interview. The reliability and validity of SCID-D-R is good-to-excellent (25, 26). The assessments were performed by trained interviewers (with B.Sc. or M.Sc. degrees).

Symptom severity was measured using the German versions of the Dissociative Experiences Scale (DES), Somatoform Dissociation Questionnaire (SDQ-20), Multidimensional Inventory of Dissociation (MID), Beck Depression Inventory (BDI), and Brief Symptoms Inventory (BSI). The DES and SDQ-20 were collected at baseline only. The scales were chosen to collect a broad range of psychiatric symptoms including dissociative symptoms that were a focus of the main study (27–29).

The DES is one of the most commonly used questionnaires measuring psychological manifestations of dissociation in typical and clinical populations (30). Ratings for the 28 items of the DES are based on an 11-point scale with increments of 10 points ranging from 0 (“never”) to 100 (“always”), with higher scores representing more frequent dissociative symptoms. Although the authors of the DES derived three factors, including absorption, amnesia, and depersonalization/derealization, results from later studies suggested a single factor only (31, 32). The DES has sound psychometric properties (30, 33–35). The psychometric properties of the German adaptation of the DES (Cronbach's alpha = 0.91; test–retest reliability Pearson *r* = 0.86; good differentiation of psychiatric patients from healthy participants, and psychiatric patients with a DD from psychiatric patients without a DD and healthy participants) are comparable to the original version (36, 37).

The SDQ-20 (38) is a 20-item rating scale that measures somatoform manifestations of dissociation such as disruptions in sensation, movement and other bodily functions. The rating of the 20 items of the SDQ-20 is based on a 5-point scale ranging from 1 to 5, yielding a minimum score of 20 and a maximum score of 100, with higher scores representing greater levels of somatoform dissociation. Factor analyses have suggested unidimensionality of the SDQ-20 (39). The psychometric properties of the SDQ-20 are good (38–40). The German adapted SDQ-20 shows excellent psychometric properties (Cronbach's alpha = 0.91; test–retest reliability Pearson *r* = 0.89; good differentiation between patients with vs. without DD) and cross-cultural validity (40).

The MID (41–43) is a comprehensive scale with 218 items (168 dissociation items, 50 validity items) for the measurement of pathological dissociation. It assesses 6 general dissociative symptoms (i.e., “memory problems,” “depersonalization,” “derealization,” “flashbacks,” “somatic symptoms,” “trance”), 11 consciously experienced intrusions from a dissociated self-state, and 6 fully-dissociated activities of another self-state. The items are rated on an 11-point scale that ranges from 0 (“never”) to 10 (“always”). The scale provides a summary score between 0 and 100 by calculating the mean score of the 168 dissociation items, multiplied by 10. The MID has demonstrated good reliability and validity (41). Preliminary data suggests sound psychometric properties of the German version of the MID (Cronbach's alphas between 0.69 and 0.94; good differentiation between patients with vs. without a Dissociative Disorder) (44).

The BDI is an internationally used questionnaire with 21 items measuring depressive symptoms. The German version of the BDI shows good reliability (45). The items are rated on a 4-point scale ranging from 0 to 3, yielding a minimum sum score of 0 and a maximum sum score of 63 with higher scores representing greater levels of depression.

The BSI is a short version of the Symptom Checklist of Derogatis (SCL-90-R). The questionnaire is internationally used and contains 53 items capturing subjective impairment due to physical and mental symptoms. It allows measurement of the dimensions “somatization,” “obsession-compulsion,” “interpersonal sensitivity,” “depression,” “anxiety,” “hostility,” “phobic anxiety,” “paranoid ideation,” and “psychoticism.” The 53 items are based on a 5-point scale ranging from 0 to 4. In this study, the global severity index score was used, consisting of the mean of the 53 items. The reliability of the German version of BSI is fair, with restricted generalizability because of a predominance of anxiety patients in the reference sample (46).

Functional impairment was assessed using the World Health Organization Disability Assessment Schedule II (WHODAS II) (47), a standardized method for measuring disability levels based on the based on the International Classification of Functioning, Disability and Health (ICF) (48). The WHODAS II has replaced the Global Assessment of Functioning Scale (GAF) (49) for describing disability associated with symptoms and signs in DSM-5. At baseline, the interviewer administered version of the WHODAS II was used; at follow-ups, the self-rating version was used. Both versions used contain 36 questions covering six domains of assessment. These are based on the International Classification of Functioning, Disability and Health (48), a system that classifies impairments in body functions and structure; activity limitations; participation restrictions; and environmental factors caused by mental or physical illness. The items are based on a 5-point rating scale with the participant rating the level of difficulty experienced as none, mild, moderate, severe, or extreme, with higher scores reflecting higher functional impairment. According to the WHODAS II manual, the participant's ratings are recorded without interpretation by the interviewers. The WHODAS II provides a total score and domain scores for the life domains “understanding and communicating” (cognition); “getting around” (mobility); “self-care” (attending to one's hygiene, dressing, eating, and staying alone); “getting along with people” (interpersonal interactions); “life activities” (domestic responsibilities, work); and “participation in society” (joining in community activities). The psychometric properties of WHODAS II are sound, with high interrater reliability (50–52).

### Data Analysis

All item scores of symptom and functional impairment measures were centered and scaled. The items of each symptom questionnaire were assigned to corresponding DSM-5 (15) symptoms. Assignment was done by the first author (JT) and reviewed by the last author (CM-P). Chi-squared tests were used to compare categorical data, *t*-tests were used to compare dimensional data between recruited participants and decliners, and study drop-outs and completers.

To investigate the temporal stability of functional impairment, we conducted a separate mixed-effects linear model (53) on WHODAS total and dimension scores for each diagnostic category. Time (baseline, 6, 12, and 18 months), age, and sex were treated as fixed effects and subject as a random effect. Similar models on symptom scores (represented by the average score across the items that were assigned to DSM-5 symptoms within a diagnostic category) were conducted to investigate temporal stability of symptoms for each diagnostic category. Cook's distance scores were calculated for each model for estimating the influence of individual observations (54). The cut-off for Cook's distance was automatically calculated using measures of internal scaling. Because there were no significant interactions involving time (data not presented), interaction terms were omitted in the models.

To investigate the association between DSM-5 symptoms and functional impairment, we conducted separate mixed-effects linear models on WHODAS total and dimension scores for each symptom. Symptom scores (represented by the mean score of the items assigned to this DSM-5 symptom), time, age, and sex were treated as fixed effects and participant as a random effect.

Age and sex were included in the mixed-effects linear regression models to control for a potential confounding effect of these subject characteristics on the results of the regression models. We have conducted a separate model for each symptom (instead of conducting one model that includes all symptoms as predictors) for two reasons: (1) we aimed to investigate the “pure” effect of each symptom on functional impairment, controlling for other influences. Including all symptoms in one model might have influenced the parameter estimates for symptoms that are correlated with each other (e.g., depressed mood and diminished interest); (2) a high number of predictors in one model might result in instability of the resulting parameter estimates. A larger sample size would have been needed to mitigate these effects. Standardized parameter estimates (β) and 95% confidence intervals (CI) were used to estimate effect sizes for the association between symptom and functional impairment.

To develop a questionnaire that predicts functional impairment, a separate penalized lasso regression model (55) was conducted on WHODAS total and dimension baseline scores. The items of the MID, DES, SDQ-20, BDI, and BSI were entered as predictors. Items with an absolute standardized estimate of greater or equal 0.5 were selected for the questionnaire. Although a change of 0.5 may be below clinical relevance, such a conservative cut-off was used to prevent excessing elimination of items based on this relatively small sample. Explained variance in predicting WHODAS total and dimension scores across 18 months by this set of items was calculated using separate mixed-effects linear models with time, age, and sex treated as additional fixed effects, and participant as a random effect.

Because the results for WHODAS dimension scores were very similar (see Supplemental Material), only WHODAS total scores are reported in the paper. A critical threshold of *p* = 0.05 (two-sided) was used; statistical analyses were performed using R V.3.4.3 (56).

## Results

### Participants

All 312 participants fulfilling study criteria were invited to participate. Of these, 136 (43.6%) declined to participate, yielding a pool of 176 recruited participants. There was no statistically significant difference between recruited participants and decliners regarding sex (*p* = 0.5), age (*p* = 0.05), and nationality (*p* = 0.9), suggesting good representativeness of our sample. Finally, data from 21 recruited participants (11.9% of the 176) were excluded from the analysis due to incomplete participation in the baseline assessment, diagnosis of a psychotic disorder (acute or remitted) or doubtful validity of the results as judged by the interviewer after discussion with the first author, e.g., suspected dissimulation or difficulties in understanding the questions. Participants with acute or stable psychotic disorder have been excluded from the study due to concerns of invalid self-reports at follow-up due to unrecognized psychotic relapse. Self-reports were completed from home without any further in-person evaluation. This procedure resulted in a final sample size of 155 participants. Participants were assessed at baseline (*N* = 155), after six (*N* = 117), 12 (*N* = 82), and 18 months (*N* = 63). Thirty participants (19.4%) had at study entrance a lifetime Axis I diagnosis, i.e., were in clinical remission; six of them had a current Axis II diagnosis. Sociodemographic and clinical characteristics at baseline and follow-up are presented in Tables 1, 2, respectively. Drop-outs did not differ significantly from study completers with respect to sociodemographic characteristics, diagnoses, and symptom severity (*p* ≥ 0.05).

**Table 1:** Sociodemographic and clinical characteristics at baseline of outpatient and daycare patients (*N* = 155).

	***N***	****%****
Female	105	67.7
Swiss nationality	110	71.0
**PRIMARY SOURCE OF INCOME**		
Own earnings	38	24.5
Earnings of partner, parents, or relatives	19	12.3
Retirement payments	3	1.9
Disability payments due to a mental disorder	30	19.4
Disability payments due to a physical disorder	6	3.9
Public welfare	35	22.6
Unemployment benefits	10	6.5
Other, e.g. savings	14	9.0
**DIAGNOSTIC CATEGORIES**^a^		
Affective disorders	78	50.3
Substance use disorders	16	10.4
Anxiety disorders	80	51.9
Somatoform disorders	15	9.7
Dissociative Disorders	30	19.4
Personality Disorders	68	44.2
	**Mean**	**SD**
Age (years)	35.8	11.8
Education (years)	12.4	3.3
Number of axis I diagnoses	1.7	1.4
DES	14.5	13.7
SDQ-20	29.7	9.9

a *According to DSM-IV; DES, Dissociative Experiences Scale; SDQ-20, Somatoform Dissociation Questionnaire*.

**Table 2:** Symptom severity and functional impairment across 18 months of outpatient and daycare patients.

	**Baseline (*****N*** **=** **155)**		**6 Months (*****N*** **=** **117)**		**12 Months (*****N*** **=** **82)**		**18 Months (*****N*** **=** **63)**	
	**Mean**	***SD***	**Mean**	***SD***	**Mean**	***SD***	**Mean**	***SD***
MID	21.0	15.9	15.8	16.0	15.1	17.1	14.9	17.2
BDI	23.0	10.5	23.1	11.7	21.6	12.7	21.7	12.6
BSI	1.3	0.7	1.3	0.8	1.2	0.8	1.1	0.8
**WHODAS II**								
Total	35.7	20.2	35.4	20.0	35.0	21.4	33.6	22.0
Understanding and Communication	31.7	22.0	33.0	21.3	32.1	23.2	31.7	22.8
Getting Around	24.2	24.6	23.5	23.6	24.2	23.6	20.8	21.9
Self-Care	17.9	20.0	17.2	20.3	20.2	22.6	18.9	21.1
Getting Along with People	40.2	30.6	46.1	29.5	46.0	30.7	43.9	31.1
Life Activities	43.3	32.6	41.7	30.3	40.9	30.4	40.4	33.4
Participation in Society	48.1	24.8	41.6	24.3	38.6	24.0	36.8	25.4

*MID, Multidimensional Inventory of Dissociation; BDI, Beck Depression Inventory; BSI, Brief Symptom Inventory; WHODAS II, World Health Organization Disability Assessment Schedule II*.

### Temporal Stability of Functional Impairment and Symptom Severity

**Figure 1:**
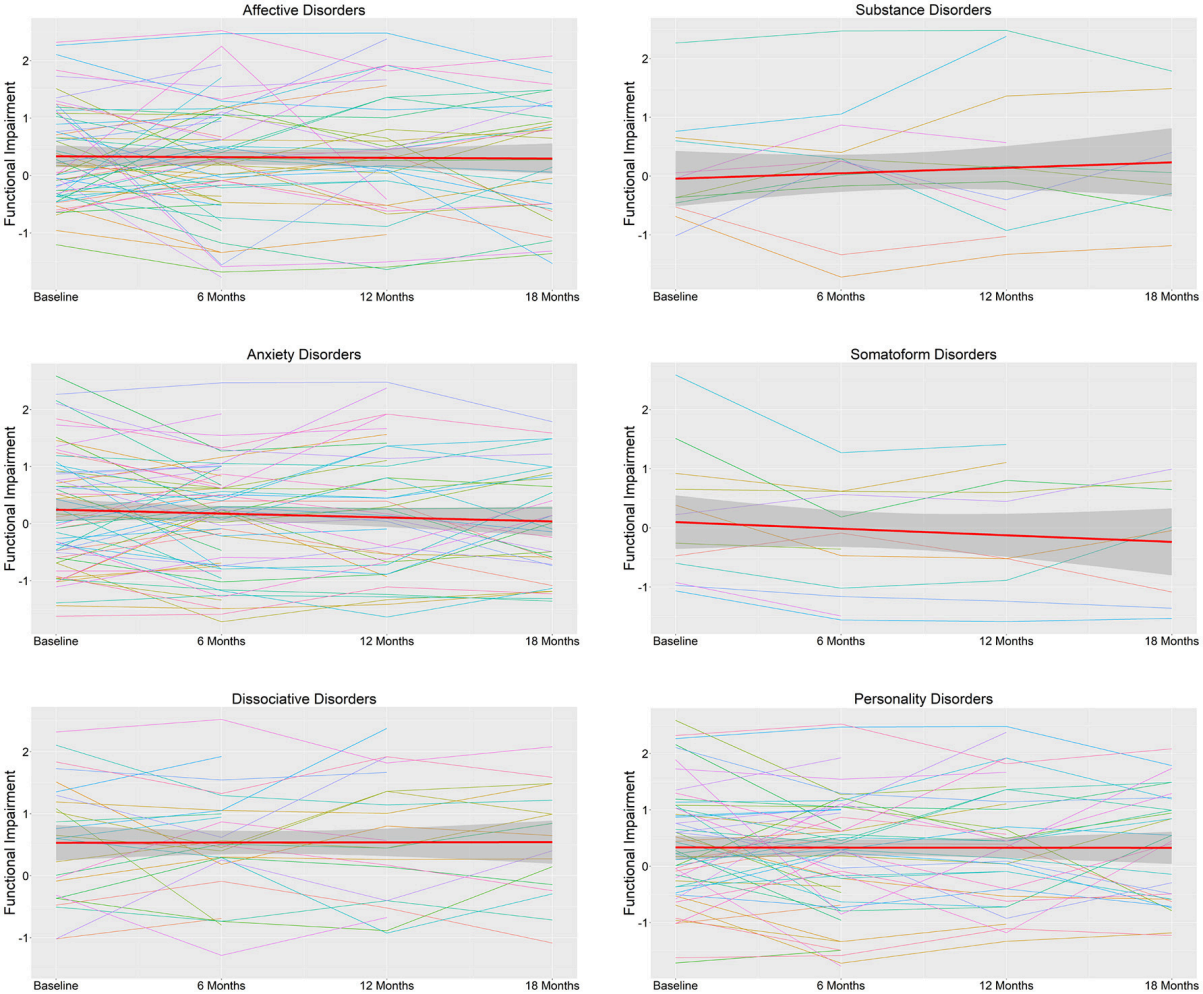
Functional impairment across WHODAS II domains during 18 months in outpatients and day-care patients (*N* = 155) by DSM-IV diagnostic category. The thick red line represents the mean functional impairment score and the associated ribbon the standard deviation. The colored thin lines represent functional impairment scores of individual cases. Higher scores represent higher impairment across life domains.

Functional impairment was stable across 18 months for participants within each diagnostic category (Figure 1), as evidenced by non-significant effects of time on mean WHODAS total scores (*p* ≥ 0.251; Table 3). There were also no significant effects of time on any of the WHODAS dimension scores (*p* ≥ 0.076; Supplementary Tables 1–6).

**Table 3:** Results of linear mixed effect models on the 18-month course of functional impairment across WHODAS II domains of outpatient and daycare patients (*N* = 155).

**Variable**	**β**	**95% CI**	**SE**	**df**	***t***	***p***
**AFFECTIVE DISORDERS (Conditional** ***R***^**2**^ **=** **0.67, Cook's distance** **=** **15/78)**						
Intercept	−0.14	−1.01, 0.74	0.45	79.21	−0.312	0.756
Time	−0.04	−0.13, 0.04	0.04	81.57	−0.969	0.335
Age	0.00	−0.02, 0.02	0.01	76.59	0.073	0.942
Sex	0.29	−0.1, 0.68	0.20	74.41	1.462	0.148
**SUBSTANCE DISORDERS (Conditional** ***R***^**2**^ **=** **0.87, Cook's distance** **=** **2/16)**						
Intercept	−1.68	−3.59, 0.23	0.97	13.86	−1.728	0.106
Time	0.02	−0.17, 0.2	0.10	8.89	0.156	0.879
Age	−0.00	−0.05, 0.04	0.02	13.12	−0.122	0.905
Sex	0.95	0.16, 1.74	0.40	13.10	2.364	0.034
**ANXIETY DISORDERS (Conditional** ***R***^**2**^ **=** **0.80, Cook's distance** **=** **14/80)**						
Intercept	−0.65	−1.66, 0.35	0.51	79.31	−1.276	0.206
Time	−0.02	−0.09, 0.05	0.03	38.07	−0.628	0.534
Age	−0.00	−0.02, 0.02	0.01	78.16	−0.127	0.899
Sex	0.53	0.08, 0.99	0.23	76.85	2.297	0.024
**SOMATOFORM DISORDERS (Conditional** ***R***^**2**^ **=** **0.88, Cook's distance** **=** **6/15)**						
Intercept	0.03	−2.69, 2.76	1.39	12.59	0.024	0.981
Time	−0.08	−0.2, 0.04	0.06	8.24	−1.236	0.251
Age	0.01	−0.03, 0.05	0.02	12.25	0.438	0.669
Sex	−0.11	−1.45, 1.22	0.68	12.36	−0.166	0.871
**DISSOCIATIVE DISORDERS (Conditional** ***R***^**2**^ **=** **0.81, Cook's distance** **=** **8/30)**						
Intercept	1.15	−0.95, 3.24	1.07	26.85	1.074	0.292
Time	0.01	−0.1, 0.12	0.06	16.01	0.240	0.813
Age	0.01	−0.01, 0.04	0.01	27.01	0.967	0.342
Sex	−0.56	−1.49, 0.38	0.48	26.54	−1.168	0.253
**PERSONALITY DISORDERS (Conditional** ***R***^**2**^ **=** **0.70, Cook's distance** **=** **10/68)**						
Intercept	−0.53	−1.63, 0.57	0.56	68.39	−0.944	0.349
Time	−0.03	-0.11, 0.06	0.04	34.75	−0.637	0.528
Age	0.00	-0.02, 0.02	0.01	68.07	0.337	0.737
Sex	0.45	-0.03, 0.92	0.24	66.27	1.848	0.069

*β, Standardized beta; diagnostic categories are related to DSM-IV. Cook's distance, Number of influential/total observations*.

**Figure 2:**
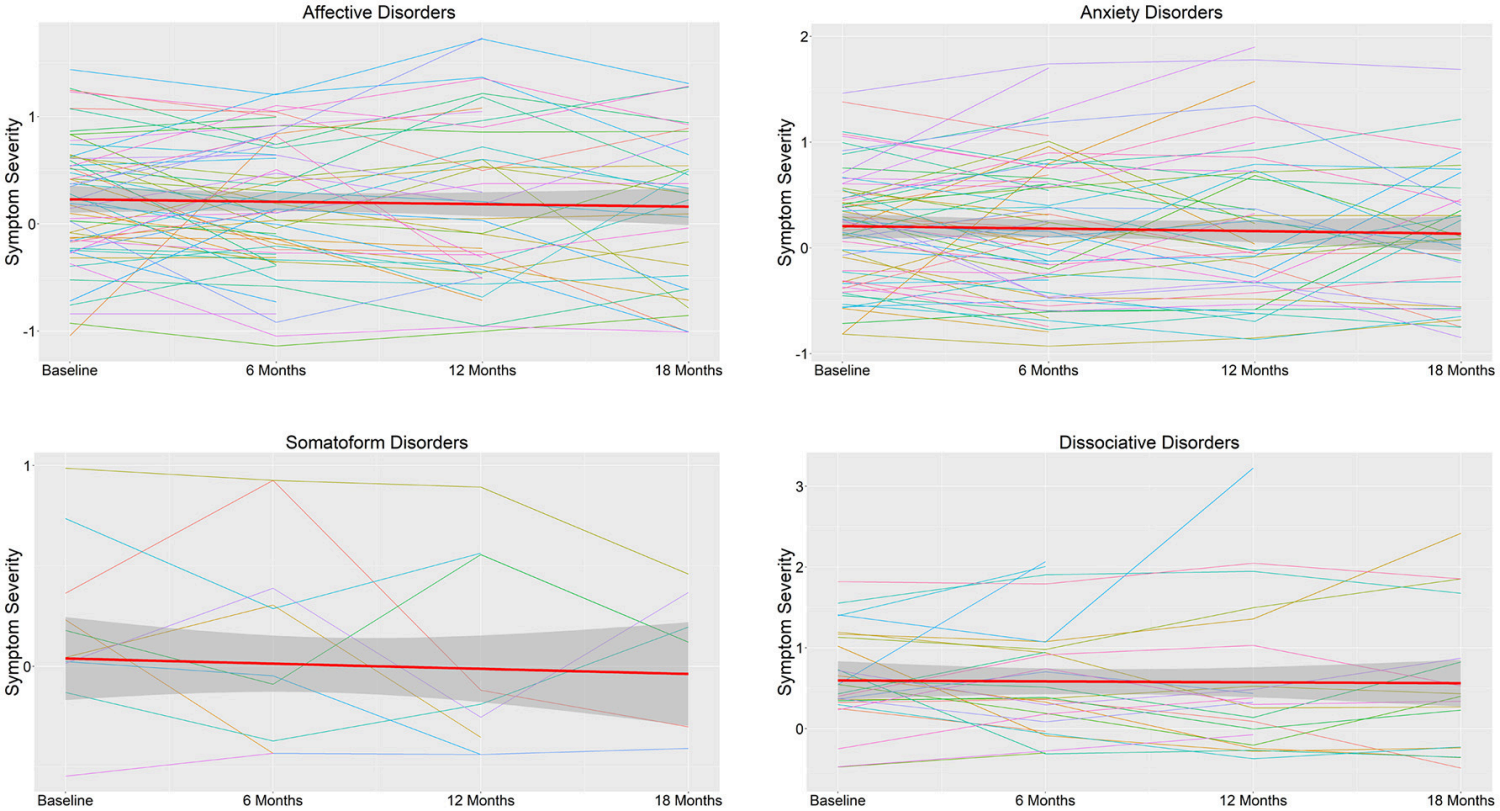
Symptom severity during 18 months in outpatients and daycare patients (*N* = 155) by DSM-IV diagnostic category. The thick red line represents the mean score across symptoms belonging to this diagnostic category and the associated ribbon the standard deviation. The colored thin lines represent the mean score across subjects of each symptom belonging to this diagnostic category. Higher scores represent higher severity.

Symptom severity related to affective disorders, anxiety disorders, somatoform disorders, and dissociative disorders was stable across 18 months (Figure 2). This was as evidenced by non-significant effects of time on mean symptom scores within each diagnostic category (*p* ≥ 0.210; Table 4).

**Table 4:** Results of linear mixed effect models on the 18-month course of symptom severity of outpatient and daycare patients (*N* = 155).

**Variable**	**β**	**95% CI**	**SE**	**df**	***t***	***P***
**AFFECTIVE DISORDERS (Conditional** ***R***^**2**^ **=** **0.75, Cook's distance** **=** **17/78)**						
Intercept	0.13	−0.45, 0.71	0.30	79.87	0.445	0.657
Time	−0.03	−0.1, 0.03	0.03	38.19	−1.028	0.310
Age	−0.00	−0.01, 0.01	0.01	76.88	−0.207	0.837
Sex	0.10	−0.15, 0.36	0.13	74.97	0.790	0.432
**ANXIETY DISORDERS (Conditional** ***R***^**2**^ **=** **0.79, Cook's distance** **=** **17/80)**						
Intercept	−0.34	−0.98, 0.3	0.33	78.49	−1.052	0.296
Time	0.00	−0.05, 0.06	0.03	30.62	0.165	0.870
Age	−0.00	−0.01, 0.01	0.01	76.74	−0.078	0.938
Sex	0.30	0.02, 0.59	0.15	75.67	2.073	0.042
**SOMATOFORM DISORDERS (Conditional** ***R***^**2**^ **=** **0.70, Cook's distance** **=** **5/15)**						
Intercept	−0.42	−1.57, 0.72	0.59	14.67	−0.724	0.480
Time	−0.06	−0.14, 0.03	0.04	11.70	−1.326	0.210
Age	0.00	−0.01, 0.02	0.01	13.62	0.194	0.849
Sex	0.25	−0.3, 0.81	0.28	14.01	0.888	0.390
**DISSOCIATIVE DISORDERS (Conditional** ***R***^**2**^ **=** **0.85, Cook's distance** **=** **7/30)**						
Intercept	0.56	−0.96, 2.07	0.77	22.66	0.717	0.481
Time	0.01	−0.1, 0.12	0.06	17.65	0.171	0.866
Age	0.00	−0.02, 0.02	0.01	24.35	0.205	0.839
Sex	−0.03	−0.7, 0.65	0.34	22.14	−0.078	0.938

*β, Standardized beta; diagnostic categories are related to DSM-IV. Cook's distance, Number of influential/total observations*.

### Association Between Symptoms and Functional Impairment

**Figure 3:**
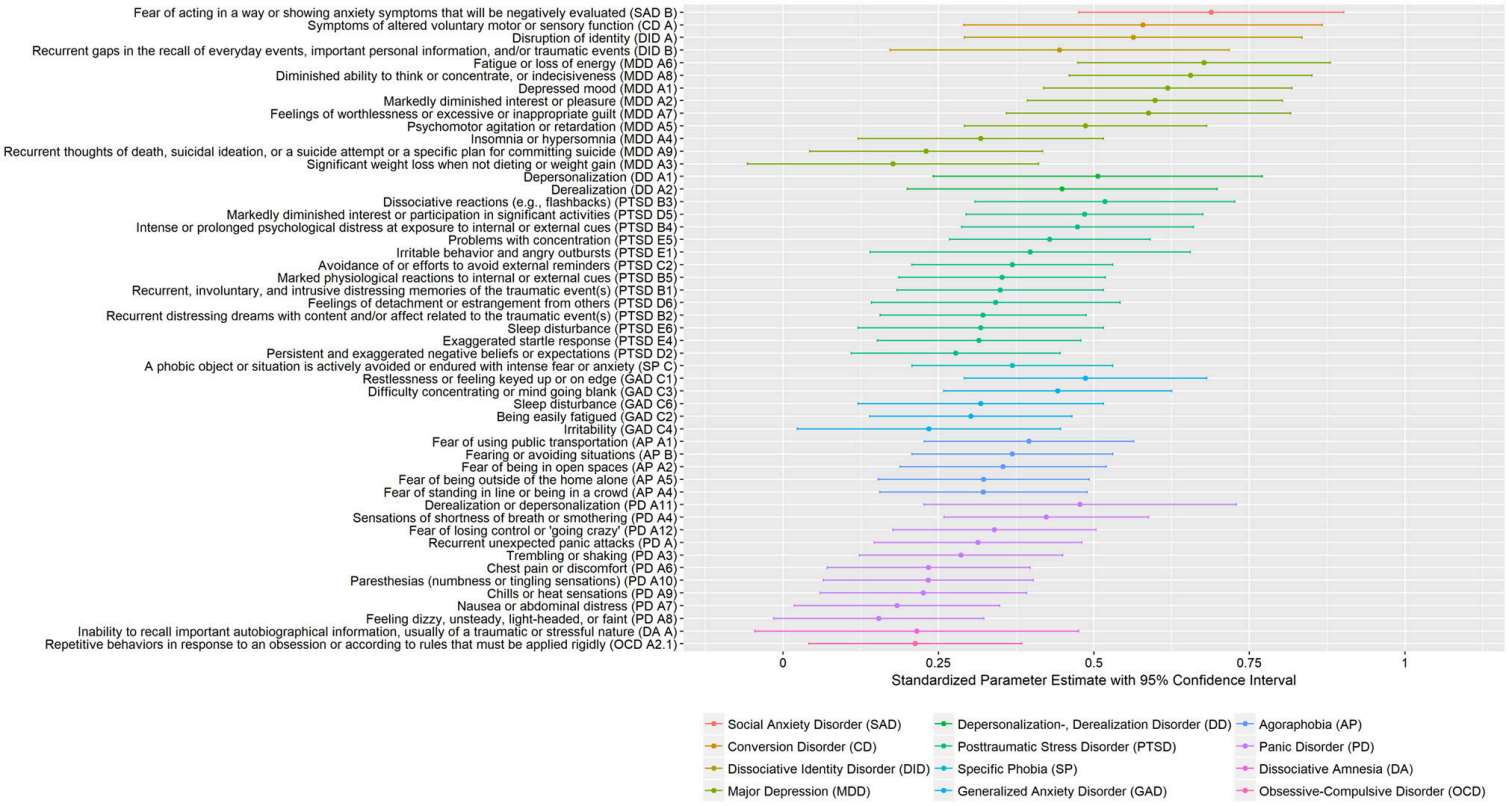
Association between DSM-5 symptoms and functional impairment across WHODAS II domains during 18 months in outpatients and daycare patients (*N* = 155). The order of the symptoms in the graph represent descending magnitudes of the mean standardized parameter estimate across the symptoms related to a diagnosis, followed by the standardized parameter estimate magnitude of each symptom related to a diagnosis. The DSM-5 symptom criterion for a symptom is given in parenthesis.

As seen in Figure 3 and Table 5, mean functional impairment effect sizes across 18 months were highest for symptoms related to social anxiety disorder, conversion disorder, dissociative identity disorder, major depression, and depersonalization/derealization disorder. Mean functional impairment effect sizes were intermediate for symptoms related to PTSD, specific phobia, generalized anxiety disorder, agoraphobia, and panic disorder. Lowest effect sizes were found for symptoms related to dissociative amnesia and obsessive-compulsive disorder. Effect sizes and 95% CIs are also presented in Supplementary Tables 7–13.

**Table 5:** Items in the Functional Impairment Prediction Scale derived from clinical and functional impairment measures of outpatients and daycare patients (*N* = 155).

**Item text**	**Original measure—item no**
Some people sometimes find that they are approached by people that they do not know who call them by another name or insist that they have met them before.	DES−6
Some people have the experience of being in a familiar place but finding it strange and unfamiliar.	DES−16
Some people find that when they are watching television or a movie they become so absorbed in the story that they are unaware of other events happening around them.	DES−17
Some people sometimes find that in certain situations they are able to do things with amazing ease and spontaneity that would usually be difficult for them (for example, sports, work, social situations, etc.).	DES−23
While watching TV, you find that you are thinking about something else.	MID−1
Feeling as if your body (or certain parts of it) are unreal.	MID−3
Having trance-like episodes where you stare off into space and lose awareness of what is going on around you.	MID−16
Thoughts being imposed on you or imposed on your mind.	MID−20
Being unable to remember your name, or age, or address.	MID−56
Being paralyzed or unable to move (for no known medical reason).	MID−60
Finding yourself lying in bed (on the sofa, etc.) with no memory of how you got there.	MID−64
Having difficulty walking (for no known medical reason).	MID−82
Hearing a lot of noise or yelling in your head.	MID−97
Re-experiencing body sensations from a past traumatic event.	MID−125
Feeling like you are “inside” yourself, watching what you are doing.	MID−133
Feeling distant or removed from your thoughts and actions.	MID−135
Reliving a past trauma so vividly that you see it, hear it, feel it, smell it, etc.	MID−145
Your thoughts and feelings are so changeable that you don't understand yourself.	MID−146
Reliving a traumatic event so totally that you think that a present-day person is actually a person from the trauma (for example, being home with your partner, suddenly reliving being raped by your alcoholic uncle, and actually thinking that your partner is your uncle—that is, you see your uncle in front of you instead of seeing your partner).	MID−156
Feeling as if there is something inside you that takes control of your behavior or speech.	MID−161
Discovering that you have a significant injury (for example, a cut, or a burn, or many bruises), and having no memory of how it happened.	MID−170
Suddenly finding yourself somewhere (for example, at the beach, at work, in a nightclub, in your car, etc.) with no memory of how you got there.	MID−173
Some thoughts are suddenly “taken away from you.”	MID−198
Feeling a struggle inside you about what to think, how to feel, what you should do.	MID−210
I dislike smells that I usually like.	SDQ−9
I cannot see for a while (as if I am blind).	SDQ−12
I grow stiff for a while.	SDQ−20
I feel sad.	BDI−1
I put off making decisions more than I used to.	BDI−13
I get tired more easily than I used to.	BDI−17
My appetite is not as good as it used to be.	BDI−18
I am worried about physical problems like aches, pains, upset stomach, or constipation.	BDI−20
I am less interested in sex than I used to be.	BDI−21
Feeling afraid in open spaces.	BSI−8
Thoughts of ending your life.	BSI−9
Feeling blocked in getting things done.	BSI−15
Feeling blue.	BSI−17
Feeling no interest in things.	BSI−18
Difficulty making decisions.	BSI−27
Feeling afraid to travel on buses, subways, or trains.	BSI−28
Trouble getting your breath.	BSI−29
Feeling weak in parts of your body.	BSI−37
Feeling tense or keyed up.	BSI−38
Feeling very self-conscious with others.	BSI−42
Never feeling close to another person.	BSI−44
Spells of terror or panic.	BSI−45
Feeling nervous when you are left alone.	BSI−47

*DES, Dissociative Experiences Scale; MID, Multidimensional Inventory of Dissociation; SDQ-20, Somatoform Dissociation Questionnaire; BDI, Beck Depression Inventory; BSI, Brief Symptom Inventory. The final questionnaire is available in the Supplemental. Instructions regarding its application can be obtained from the corresponding author*.

With very few exceptions, all symptoms had a statistically significant effect on functioning. However, there was often high variability in the strength of the association between the various symptoms of a specific disorder and functional impairment. In major depression, fatigue or loss of energy, thinking or concentration problems, and depressed mood were significantly higher than recurrent thoughts of death, suicidality, weight loss or weight gain. Although statistically not significant, sleeping problems seem to have a smaller effect on functioning than diminished interest or pleasure, feelings of worthlessness, guilt, and psychomotor agitation or retardation.

Among symptoms in posttraumatic stress disorder, dissociate reactions (e.g., flashbacks), diminished interest or participation in significant activities, and psychological distress at exposure to traumatic cues, had the strongest effects on functioning. In contrast, exaggerated startle responses and cognitive distortions had small effect sizes.

In panic disorder, derealization or depersonalization and breathing problems seem to have stronger effects on functioning than other mental and physical manifestations of fear. The observed differences may have occurred by chance, as indicated by the overlapping confidence intervals.

### Questionnaire Items That Predict Functional Impairment

Of all 290 symptom items in the MID, DES, SDQ-20, BDI, and BSI, 47 predicted functional impairment (Table 5). Among the 47 items, 27 items referred to various type of dissociative experiences, 11 items to depressive symptoms, and 9 items to symptoms of anxiety. Explained variance (conditional *R*^2^) of this final set of items was 0.77 for predicting WHODAS scores across domains, 0.68 for “understanding and communicating,” 0.62 for “getting around,” 0.64 for “self-care,” 0.74 for “getting along with people,” 0.72 for “life activities” and 0.68 for “participation in society”.

## Discussion

The main aim of this prospective study was to investigate the temporal stability of functional impairments and their association with psychiatric signs and symptoms. We found high stability in the extent of functional impairment across 18 months. Substantial relative differences were observed in the strength of the association of DSM-5 signs and symptoms and functional impairment measures both between and within disorders. Core symptoms of social anxiety disorder, major depression, conversion disorder and dissociative identity disorder had among the strongest relative effects on functioning.

Average levels of functional impairment in our sample remained stable across 18 months and diagnostic categories. This was accompanied by stable average levels of symptoms related to affective disorders, anxiety disorders, somatoform disorder, and dissociative disorders. Although symptom levels in major depression fluctuate over time, their course is often chronic (57). The same is true for generalized anxiety disorders (58) and PTSD (59). While some studies found that disability varies directly with the levels of depressive symptoms (60), others observed long disability even after remission of depressive symptoms (61).

Our finding that fear of negative evaluations (a core symptom in social anxiety disorder) has the highest adverse influence on the course of function across life domains supports a recent meta-analysis on anxiety disorders and functional impairment that found the highest correlation between global functioning and social anxiety disorder (9). Their observation of a high correlation of obsessive-compulsive disorder with global functioning contrasts with our finding of a relatively low effect of compulsive behavior on functioning. It might be that impaired functioning is primarily driven by obsessive thoughts and not compulsive behaviors. This finding needs to be explored further in future studies because we did not collect data regarding obsessive thoughts, and the meta-analysis did not discriminate between distinct symptoms of obsessive-compulsive disorder.

The relatively higher effect sizes we observed for symptoms of major depression compared to symptoms of anxiety disorders other than social anxiety is in line with previous evidence from primary care settings (62). Beyond that, our results suggest that disability in major depression may be primarily driven by fatigue, mood, and cognitive impairments and less by sleeping problems, suicidality or appetite/weight changes. Similarly, we found that PTSD signs and symptoms (i.e., dissociative reactions, diminished interest or participation, psychological distress at exposure to trauma cues, and concentration problems) from three DSM-5 clusters (B, D, E) showed the highest association with functional impairment. This might explain inconsistencies in previous studies that investigated how PTSD clusters, but not distinct symptoms in the clusters, were associated with impaired functioning (63–65).

Four of the 10 symptoms that had the highest impact on functioning in our study (i.e., altered voluntary or sensory function, disruption of identity, flashbacks, and depersonalization) related to dissociation. This finding supports previous evidence showing the profound influence these symptoms may have on functioning (27, 66, 67). This relationship seems particularly relevant given the high scrutiny individuals with these disorders often encounter in the context of insurance medicine (68).

The 47 symptom items of the Functional Impairment Prediction Scale (FIPS), that we developed in this study, mostly refer to dissociative and depressive symptoms. This does not seem surprising given the high association we observed between functional impairment across life domains and DSM-5 symptoms related to conversion disorder, dissociative identity disorder and major depression. Although anxiety symptom items in the FIPS were weaker associated with WHODAS total scores, they predicted functioning levels in specific life domains, e.g., mobility. We are not aware of any empirically developed measure that allows prediction of functional impairment from symptom profiles. Hence, the FIPS may be a valuable tool in legal assessments. The scale can add an additional level of empirically derived evidence to the evaluation of working capacity. Moreover, the combined application of the FIPS and WHODAS II would allow to compare predicted and self-reported functional impairment, thereby contributing to the evaluation of consistency in legal reports. It is noteworthy, however, that the item selection of the FIPS need be considered as preliminary and requires validation in separate samples. The 47 questions used in FIPS and their rating scales are presented in Supplementary Table 14.

The findings from our study have several implications for legal assessments and rehabilitation. Medical experts in insurance medicine should be aware that psychiatric disability has a strong tendency to persist for long periods. Consequently, they should be realistic when considering the chances for functional improvement under treatment. Overly optimistic prognostication may undermine a claimant's entitlement to receive occupational disability benefits. Moreover, assessors should recommend treatment modalities that target signs and symptoms with strong influences on work functioning. Concentration problems showed the fourth-highest association with impaired function among PTSD symptoms in our study. Therefore, if a traumatized claimant has severe concentration problems and lower levels of traumatic re-experiencing, a treatment plan could benefit from inclusion of attention training (69), even if trauma exposure therapy is considered the standard treatment in PTSD (70). Finally, our results enhance the empirical basis for the work capacity evaluation in legal assessments, which is urgently required in insurance medicine (71). For instance, arguing an inability to work due to sleeping problems in the absence of other relevant symptoms would be difficult to justify given the relatively low association we observed between sleeping disturbances and impaired functioning.

A strength of this study is the employment of a detailed and rigorous diagnostic characterization that included SCID-I, SCID-II, and SCID-D-R interviews for every participant enrolled. The use of consecutive recruitment by service providers allows better generalization of our findings to the population of general psychiatric patients seeking treatment. Eligible patients who refused to participate in this study and drop-outs are a potential threat to the generalizability of the results. However, study refusers and drop-outs did not differ in sociodemographic or clinical characteristics from study participants and study completers, respectively, which makes a recruitment and/or drop-out bias unlikely. Data presented in this paper have been retrieved as part of a study on global functioning in dissociative disorders (27–29), which has influenced the selection of the scales. The scales used in the study represent major, but not all, areas of psychopathology in general psychiatric outpatients. Consequently, we cannot preclude that there are other signs and symptoms which have contributed to functional impairment in this study. Given the low sample size in some diagnostic categories (e.g., substance disorder and somatoform disorder), some weaker associations with work disability may not have been detected. A positive association between signs, symptoms and functional measures does not necessarily imply causal relationships among them. Ultimately, causality in psychiatric disability needs to be investigated in experimental studies. Finally, the Functional Impairment Prediction Scale that was developed in this study needs to be cross-validated in an independent sample of psychiatric patients.

Taken together, this study provides novel insights concerning which signs and symptoms may be associated with functional impairment in mental disorders. Given the capability for somatic and mental symptoms associated with social anxiety, depression, and dissociation to predict future disability, these measures have strong potential for guiding rehabilitation planning and prognostic evaluation in insurance medicine. The high temporal stability of functional impairment also calls for therapeutic interventions, such as functional training (72), that go beyond the treatment of psychopathological symptoms. Confirmation of the validity of the Functional Impairment Prediction Scale in predicting disability in future studies will foster the scale as a valuable, empirical-based extension in legal assessments of working capacity.

## Author's Note

The work was carried out at the Psychiatric Services of the County of St. Gallen-North, Zurcherstrasse 30, 9500 Wil, Switzerland.

## Author Contributions

CM-P and MR designed the study that provided the data for this paper. CM-P, DW, and NP collected the data. JT, TZ, and CM-P conducted the analyses and wrote the paper.

### Conflict of Interest Statement

The authors declare that the research was conducted in the absence of any commercial or financial relationships that could be construed as a potential conflict of interest.
