# Supplementary material for: Psychiatric Symptom Profiles Predict Functional Impairment
Source: Front Psychiatry. 2019 Feb 13;10:37. doi: 10.3389/fpsyt.2019.00037 (PMC6396718; doi:10.3389/fpsyt.2019.00037)
Supplement: Supplementary file 1 [file Data_Sheet_1.docx]

Supplementary Material

Psychiatric Symptom Profiles Predict Functional Impairment

Joachim Tanner^1^, Thomas Zeffiro^1,2,3^, Daniela Wyss^4^, Noelle Perron^4,5^, Michel Rufer^6^, Christoph Mueller-Pfeiffer^1,4*^

^1^Department of Consultation-Liaison-Psychiatry and Psychosomatic Medicine, University Hospital Zurich, University of Zurich, Zurich, Switzerland

^2^Neurometrika, Potomac, MD, USA

^3^Department of Diagnostic Radiology and Nuclear Medicine, University of Maryland, Baltimore, MD, USA

^4^Center of Education and Research (COEUR), Psychiatric Services of the County of St. Gallen-North, Wil, Switzerland

^5^Clinical Psychology and Psychotherapy, University of Bern, Bern, Switzerland

^6^Department of Psychiatry, Psychotherapy, and Psychosomatics, Psychiatric Hospital Zurich, University of Zurich, Zurich, Switzerland

*** Correspondence:**Christoph Mueller-Pfeiffer
University Hospital of Zurich, Consultation-Liaison-Psychiatry and Psychosomatic Medicine, Culmannstrasse 8, 8091 Zurich, Phone: +41 44 255 52 80, Fax: +41 44 255 44 08, E-mail: christoph.mueller-pfeiffer@access.uzh.ch

# Supplementary Figures and Tables

## Supplementary Tables

**Supplementary Table 1.** Results of linear mixed effect models on the 18-month course of functional impairment in the WHODAS II domain 'Understanding and Communication' in out- and day care-patients (*N* = 155)

| Variable | β | 95% CI | SE | df | *t* | *p* |
| --- | --- | --- | --- | --- | --- | --- |
| Affective Disorders (Conditional RP^2^P = 0.64) | | | | | | |
| Intercept | 0.04 | -0.93, 1.01 | 0.49 | 79.17 | 0.089 | 0.930 |
| Time | -0.06 | -0.15, 0.04 | 0.05 | 99.82 | -1.200 | 0.233 |
| Age | -0.00 | -0.02, 0.02 | 0.01 | 76.55 | -0.265 | 0.792 |
| Sex | 0.24 | -0.19, 0.66 | 0.22 | 74.05 | 1.087 | 0.281 |
| Substance Disorders (Conditional RP^2^P = 0.71) | | | | | | |
| Intercept | -0.79 | -2.92, 1.33 | 1.08 | 14.26 | -0.732 | 0.476 |
| Time | 0.06 | -0.12, 0.25 | 0.10 | 22.48 | 0.670 | 0.510 |
| Age | -0.00 | -0.05, 0.05 | 0.03 | 13.22 | -0.042 | 0.968 |
| Sex | 0.47 | -0.4, 1.35 | 0.45 | 13.19 | 1.061 | 0.308 |
| Anxiety Disorders (Conditional RP^2^P = 0.69) | | | | | | |
| Intercept | -0.33 | -1.35, 0.7 | 0.52 | 80.90 | -0.620 | 0.537 |
| Time | -0.02 | -0.11, 0.07 | 0.04 | 43.72 | -0.425 | 0.673 |
| Age | 0.00 | -0.02, 0.02 | 0.01 | 79.02 | 0.162 | 0.871 |
| Sex | 0.27 | -0.19, 0.73 | 0.24 | 76.95 | 1.156 | 0.251 |
| Somatoform Disorders (Conditional RP^2^P = 0.82) | | | | | | |
| Intercept | -0.34 | -2.98, 2.29 | 1.35 | 13.91 | -0.256 | 0.802 |
| Time | -0.08 | -0.32, 0.17 | 0.12 | 11.90 | -0.622 | 0.546 |
| Age | 0.01 | -0.03, 0.04 | 0.02 | 12.29 | 0.454 | 0.658 |
| Sex | 0.05 | -1.22, 1.33 | 0.65 | 13.10 | 0.084 | 0.935 |
| Dissociative Disorders (Conditional RP^2^P = 0.71) | | | | | | |
| Intercept | 1.32 | -0.83, 3.47 | 1.10 | 26.87 | 1.204 | 0.239 |
| Time | -0.06 | -0.19, 0.06 | 0.06 | 19.32 | -1.004 | 0.328 |
| Age | 0.01 | -0.02, 0.04 | 0.01 | 27.17 | 0.771 | 0.447 |
| Sex | -0.55 | -1.51, 0.4 | 0.49 | 26.02 | -1.136 | 0.266 |
| Personality Disorders (Conditional RP^2^P = 0.61) | | | | | | |
| Intercept | -0.13 | -1.23, 0.98 | 0.56 | 70.99 | -0.227 | 0.821 |
| Time | -0.01 | -0.11, 0.08 | 0.05 | 118.01 | -0.261 | 0.794 |
| Age | -0.00 | -0.02, 0.02 | 0.01 | 70.47 | -0.249 | 0.804 |
| Sex | 0.27 | -0.21, 0.74 | 0.24 | 67.90 | 1.107 | 0.272 |
| β: Standardized beta; diagnostic categories are related to DSM-IV. | | | | | | |

**Supplementary Table 2.** Results of linear mixed effect models on the 18-month course of functional impairment in the WHODAS II domain 'Getting Around' in out- and day care-patients (*N* = 155)

| Variable | β | 95% CI | SE | df | *t* | *p* |
| --- | --- | --- | --- | --- | --- | --- |
| Affective Disorders (Conditional RP^2^P = 0.50) | | | | | | |
| Intercept | -0.69 | -1.59, 0.2 | 0.46 | 84.43 | -1.524 | 0.131 |
| Time | 0.03 | -0.07, 0.13 | 0.05 | 108.25 | 0.593 | 0.555 |
| Age | 0.01 | -0.01, 0.03 | 0.01 | 79.85 | 0.969 | 0.335 |
| Sex | 0.28 | -0.11, 0.67 | 0.20 | 76.10 | 1.397 | 0.166 |
| Substance Disorders (Conditional RP^2^P = 0.75) | | | | | | |
| Intercept | -0.82 | -2.62, 0.99 | 0.92 | 15.37 | -0.887 | 0.389 |
| Time | 0.07 | -0.17, 0.3 | 0.12 | 11.37 | 0.563 | 0.584 |
| Age | -0.03 | -0.07, 0.01 | 0.02 | 13.53 | -1.306 | 0.213 |
| Sex | 0.88 | 0.16, 1.61 | 0.37 | 13.54 | 2.379 | 0.033 |
| Anxiety Disorders (Conditional RP^2^P = 0.62) | | | | | | |
| Intercept | -1.49 | -2.44, -0.54 | 0.48 | 79.83 | -3.068 | 0.003 |
| Time | 0.06 | -0.03, 0.14 | 0.04 | 44.32 | 1.305 | 0.199 |
| Age | 0.01 | 0, 0.03 | 0.01 | 77.78 | 1.706 | 0.092 |
| Sex | 0.58 | 0.16, 1.01 | 0.22 | 74.95 | 2.674 | 0.009 |
| Somatoform Disorders (Conditional RP^2^P = 0.83) | | | | | | |
| Intercept | -1.27 | -3.84, 1.29 | 1.31 | 12.76 | -0.974 | 0.348 |
| Time | -0.03 | -0.16, 0.11 | 0.07 | 8.99 | -0.412 | 0.690 |
| Age | 0.02 | -0.01, 0.06 | 0.02 | 11.93 | 1.294 | 0.220 |
| Sex | 0.21 | -1.04, 1.46 | 0.64 | 12.33 | 0.330 | 0.747 |
| Dissociative Disorders (Conditional RP^2^P = 0.54) | | | | | | |
| Intercept | 0.10 | -1.96, 2.15 | 1.05 | 25.08 | 0.093 | 0.927 |
| Time | 0.10 | -0.05, 0.24 | 0.07 | 20.80 | 1.330 | 0.198 |
| Age | 0.01 | -0.02, 0.04 | 0.01 | 25.64 | 0.718 | 0.479 |
| Sex | -0.15 | -1.05, 0.76 | 0.46 | 23.70 | -0.315 | 0.755 |
| Personality Disorders (Conditional RP^2^P = 0.58) | | | | | | |
| Intercept | -1.13 | -2.21, -0.04 | 0.55 | 71.10 | -2.029 | 0.046 |
| Time | 0.01 | -0.09, 0.11 | 0.05 | 42.44 | 0.190 | 0.850 |
| Age | 0.01 | 0, 0.03 | 0.01 | 70.49 | 1.447 | 0.152 |
| Sex | 0.49 | 0.02, 0.95 | 0.24 | 67.10 | 2.053 | 0.044 |
| β: Standardized beta; diagnostic categories are related to DSM-IV. | | | | | | |

**Supplementary Table 3.** Results of linear mixed effect models on the 18-month course of functional impairment in the WHODAS II domain 'Self-Care' in out- and day care-patients (*N* = 155)

| Variable | β | 95% CI | SE | df | *t* | *p* |
| --- | --- | --- | --- | --- | --- | --- |
| Affective Disorders (Conditional RP^2^P = 0.57) | | | | | | |
| Intercept | -0.43 | -1.44, 0.58 | 0.52 | 85.91 | -0.834 | 0.406 |
| Time | -0.02 | -0.11, 0.08 | 0.05 | 146.98 | -0.332 | 0.740 |
| Age | 0.00 | -0.02, 0.02 | 0.01 | 81.99 | 0.369 | 0.713 |
| Sex | 0.31 | -0.13, 0.75 | 0.22 | 78.60 | 1.363 | 0.177 |
| Substance Disorders (Conditional RP^2^P = 0.87) | | | | | | |
| Intercept | -0.44 | -2.68, 1.81 | 1.14 | 14.40 | -0.380 | 0.709 |
| Time | 0.07 | -0.19, 0.32 | 0.13 | 10.26 | 0.511 | 0.620 |
| Age | -0.03 | -0.08, 0.03 | 0.03 | 13.33 | -0.925 | 0.371 |
| Sex | 0.68 | -0.24, 1.6 | 0.47 | 13.38 | 1.454 | 0.169 |
| Anxiety Disorders (Conditional RP^2^P = 0.68) | | | | | | |
| Intercept | -0.70 | -1.69, 0.29 | 0.51 | 82.96 | -1.390 | 0.168 |
| Time | -0.03 | -0.11, 0.06 | 0.04 | 40.66 | -0.632 | 0.531 |
| Age | -0.01 | -0.02, 0.01 | 0.01 | 81.02 | -0.958 | 0.341 |
| Sex | 0.68 | 0.23, 1.12 | 0.23 | 79.01 | 2.980 | 0.004 |
| Somatoform Disorders (Conditional RP^2^P = 0.65) | | | | | | |
| Intercept | -0.98 | -4.48, 2.52 | 1.78 | 14.53 | -0.549 | 0.591 |
| Time | -0.11 | -0.36, 0.14 | 0.13 | 32.47 | -0.878 | 0.386 |
| Age | 0.00 | -0.04, 0.05 | 0.02 | 13.50 | 0.164 | 0.872 |
| Sex | 0.72 | -0.98, 2.42 | 0.87 | 13.88 | 0.832 | 0.419 |
| Dissociative Disorders (Conditional RP^2^P = 0.59) | | | | | | |
| Intercept | 2.29 | 0.1, 4.49 | 1.12 | 26.32 | 2.048 | 0.051 |
| Time | -0.02 | -0.17, 0.14 | 0.08 | 20.38 | -0.208 | 0.838 |
| Age | 0.00 | -0.03, 0.03 | 0.02 | 26.77 | 0.246 | 0.807 |
| Sex | -0.90 | -1.87, 0.07 | 0.49 | 25.06 | -1.818 | 0.081 |

| **Supplementary Table 3.** Results of linear mixed effect models on the 18-month course of Personality Disorders (Conditional RP^2^P = 0.65) | | | | | | |
| --- | --- | --- | --- | --- | --- | --- |
| Intercept | -0.20 | -1.33, 0.93 | 0.58 | 74.91 | -0.345 | 0.731 |
| Time | -0.06 | -0.15, 0.04 | 0.05 | 40.22 | -1.151 | 0.256 |
| Age | -0.01 | -0.03, 0.01 | 0.01 | 74.60 | -0.770 | 0.444 |
| Sex | 0.45 | -0.03, 0.93 | 0.25 | 70.95 | 1.831 | 0.071 |
| β: Standardized beta; diagnostic categories are related to DSM-IV. | | | | | | |

**Supplementary Table 4.** Results of linear mixed effect models on the 18-month course of functional impairment in the WHODAS II domain 'Getting Along with People' in out- and day care-patients (*N* = 155)

| Variable | β | 95% CI | SE | df | *t* | *p* |
| --- | --- | --- | --- | --- | --- | --- |
| Affective Disorders (Conditional RP^2^P = 0.68) | | | | | | |
| Intercept | -0.08 | -0.99, 0.83 | 0.47 | 79.10 | -0.172 | 0.864 |
| Time | -0.03 | -0.13, 0.06 | 0.05 | 42.42 | -0.700 | 0.487 |
| Age | -0.01 | -0.02, 0.01 | 0.01 | 76.31 | -0.703 | 0.484 |
| Sex | 0.38 | -0.02, 0.78 | 0.21 | 73.94 | 1.839 | 0.070 |
| Substance Disorders (Conditional RP^2^P = 0.77) | | | | | | |
| Intercept | -2.55 | -4.04, -1.07 | 0.76 | 15.13 | -3.367 | 0.004 |
| Time | 0.05 | -0.15, 0.26 | 0.10 | 8.65 | 0.484 | 0.640 |
| Age | 0.03 | 0, 0.07 | 0.02 | 13.41 | 1.883 | 0.082 |
| Sex | 0.80 | 0.2, 1.41 | 0.31 | 13.32 | 2.615 | 0.021 |
| Anxiety Disorders (Conditional RP^2^P = 0.76) | | | | | | |
| Intercept | -0.30 | -1.28, 0.68 | 0.50 | 79.73 | -0.607 | 0.545 |
| Time | -0.03 | -0.1, 0.05 | 0.04 | 45.72 | -0.664 | 0.510 |
| Age | -0.01 | -0.03, 0.01 | 0.01 | 78.35 | -1.085 | 0.281 |
| Sex | 0.51 | 0.07, 0.95 | 0.22 | 76.09 | 2.289 | 0.025 |
| Somatoform Disorders (Conditional RP^2^P = 0.86) | | | | | | |
| Intercept | 0.94 | -1.33, 3.2 | 1.15 | 13.67 | 0.811 | 0.431 |
| Time | -0.13 | -0.26, -0.01 | 0.07 | 7.64 | -2.051 | 0.076 |
| Age | 0.00 | -0.03, 0.03 | 0.02 | 13.08 | 0.020 | 0.984 |
| Sex | -0.53 | -1.63, 0.57 | 0.56 | 13.31 | -0.941 | 0.363 |
| Dissociative Disorders (Conditional RP^2^P = 0.73) | | | | | | |
| Intercept | -0.02 | -2.21, 2.18 | 1.12 | 27.18 | -0.016 | 0.987 |
| Time | 0.06 | -0.05, 0.16 | 0.05 | 18.04 | 1.026 | 0.318 |
| Age | -0.00 | -0.03, 0.02 | 0.02 | 27.32 | -0.330 | 0.744 |
| Sex | 0.24 | -0.74, 1.22 | 0.50 | 26.75 | 0.480 | 0.635 |
| Personality Disorders (Conditional RP^2^P = 0.60) | | | | | | |
| Intercept | -0.53 | -1.48, 0.41 | 0.48 | 67.93 | -1.106 | 0.273 |
| Time | -0.04 | -0.13, 0.04 | 0.04 | 101.25 | -0.943 | 0.348 |
| Age | 0.01 | -0.01, 0.02 | 0.01 | 67.35 | 0.867 | 0.389 |
| Sex | 0.47 | 0.06, 0.87 | 0.21 | 64.56 | 2.268 | 0.027 |
| β: Standardized beta; diagnostic categories are related to DSM-IV. | | | | | | |

**Supplementary Table 5.** Results of linear mixed effect models on the 18-month course of functional impairment in the WHODAS II domain 'Life Activities' in out- and day care-patients (*N* = 155)

| Variable | β | 95% CI | SE | df | *t* | *p* |
| --- | --- | --- | --- | --- | --- | --- |
| Affective Disorders (Conditional RP^2^P = 0.64) | | | | | | |
| Intercept | -0.01 | -0.97, 0.96 | 0.49 | 77.60 | -0.012 | 0.991 |
| Time | -0.07 | -0.16, 0.02 | 0.04 | 44.89 | -1.594 | 0.118 |
| Age | 0.00 | -0.01, 0.02 | 0.01 | 74.86 | 0.495 | 0.622 |
| Sex | 0.08 | -0.35, 0.51 | 0.22 | 71.99 | 0.371 | 0.712 |
| Substance Disorders (Conditional RP^2^P = 0.80) | | | | | | |
| Intercept | -0.55 | -2.45, 1.34 | 0.97 | 14.47 | -0.575 | 0.574 |
| Time | -0.05 | -0.23, 0.14 | 0.10 | 12.52 | -0.491 | 0.632 |
| Age | -0.03 | -0.07, 0.02 | 0.02 | 13.20 | -1.142 | 0.274 |
| Sex | 0.77 | 0, 1.54 | 0.39 | 13.23 | 1.949 | 0.073 |
| Anxiety Disorders (Conditional RP^2^P = 0.73) | | | | | | |
| Intercept | -0.11 | -1.14, 0.92 | 0.53 | 78.64 | -0.204 | 0.839 |
| Time | -0.04 | -0.11, 0.04 | 0.04 | 45.81 | -0.894 | 0.376 |
| Age | -0.00 | -0.02, 0.01 | 0.01 | 77.22 | -0.491 | 0.625 |
| Sex | 0.24 | -0.22, 0.71 | 0.24 | 75.88 | 1.026 | 0.308 |
| Somatoform Disorders (Conditional RP^2^P = 0.84) | | | | | | |
| Intercept | 1.53 | -1.23, 4.29 | 1.41 | 12.93 | 1.086 | 0.297 |
| Time | -0.07 | -0.24, 0.11 | 0.09 | 15.96 | -0.750 | 0.464 |
| Age | -0.01 | -0.05, 0.03 | 0.02 | 12.77 | -0.304 | 0.766 |
| Sex | -0.56 | -1.92, 0.79 | 0.69 | 12.69 | -0.816 | 0.429 |
| Dissociative Disorders (Conditional RP^2^P = 0.81) | | | | | | |
| Intercept | 1.72 | -0.41, 3.85 | 1.09 | 26.95 | 1.584 | 0.125 |
| Time | -0.09 | -0.21, 0.02 | 0.06 | 23.56 | -1.606 | 0.122 |
| Age | 0.01 | -0.02, 0.04 | 0.01 | 27.00 | 0.907 | 0.372 |
| Sex | -0.77 | -1.72, 0.17 | 0.48 | 26.42 | -1.600 | 0.121 |
| Personality Disorders (Conditional RP^2^P = 0.70) | | | | | | |
| Intercept | -0.10 | -1.23, 1.03 | 0.58 | 67.27 | -0.175 | 0.862 |
| Time | -0.07 | -0.17, 0.04 | 0.05 | 46.63 | -1.213 | 0.231 |
| Age | -0.00 | -0.02, 0.02 | 0.01 | 66.32 | -0.230 | 0.819 |
| Sex | 0.22 | -0.26, 0.71 | 0.25 | 63.82 | 0.897 | 0.373 |
| β: Standardized beta; diagnostic categories are related to DSM-IV. | | | | | | |

**Supplementary Table 6.** Results of linear mixed effect models on the 18-month course of functional impairment in the WHODAS II domain 'Participation in Society' in out- and day care-patients (*N* = 155)

| Variable | β | 95% CI | SE | df | *t* | *p* |
| --- | --- | --- | --- | --- | --- | --- |
| Affective Disorders (Conditional RP^2^P = 0.53) | | | | | | |
| Intercept | -0.34 | -1.14, 0.46 | 0.41 | 82.47 | -0.840 | 0.403 |
| Time | -0.05 | -0.15, 0.04 | 0.05 | 82.86 | -1.126 | 0.264 |
| Age | 0.01 | -0.01, 0.02 | 0.01 | 77.86 | 0.663 | 0.509 |
| Sex | 0.34 | -0.01, 0.69 | 0.18 | 74.32 | 1.927 | 0.058 |
| Substance Disorders (Conditional RP^2^P = 0.82) | | | | | | |
| Intercept | -2.19 | -3.94, -0.44 | 0.90 | 13.82 | -2.446 | 0.028 |
| Time | -0.02 | -0.2, 0.17 | 0.10 | 7.96 | -0.177 | 0.864 |
| Age | 0.02 | -0.03, 0.06 | 0.02 | 12.73 | 0.708 | 0.492 |
| Sex | 0.89 | 0.17, 1.61 | 0.37 | 12.74 | 2.413 | 0.032 |
| Anxiety Disorders (Conditional RP^2^P = 0.70) | | | | | | |
| Intercept | -0.86 | -1.82, 0.1 | 0.49 | 81.68 | -1.763 | 0.082 |
| Time | -0.05 | -0.13, 0.03 | 0.04 | 43.24 | -1.186 | 0.242 |
| Age | 0.00 | -0.02, 0.02 | 0.01 | 80.02 | 0.066 | 0.947 |
| Sex | 0.64 | 0.2, 1.07 | 0.22 | 77.66 | 2.890 | 0.005 |
| Somatoform Disorders (Conditional RP^2^P = 0.84) | | | | | | |
| Intercept | 0.08 | -2.58, 2.74 | 1.36 | 12.60 | 0.059 | 0.954 |
| Time | 0.03 | -0.15, 0.2 | 0.09 | 8.35 | 0.317 | 0.759 |
| Age | 0.00 | -0.03, 0.04 | 0.02 | 12.22 | 0.247 | 0.809 |
| Sex | -0.11 | -1.41, 1.19 | 0.66 | 12.25 | -0.165 | 0.872 |
| Dissociative Disorders (Conditional RP^2^P = 0.72) | | | | | | |
| Intercept | 0.02 | -1.81, 1.85 | 0.93 | 26.67 | 0.019 | 0.985 |
| Time | 0.04 | -0.08, 0.16 | 0.06 | 18.86 | 0.607 | 0.551 |
| Age | 0.02 | 0, 0.05 | 0.01 | 26.73 | 1.808 | 0.082 |
| Sex | -0.21 | -1.02, 0.6 | 0.41 | 25.85 | -0.504 | 0.619 |
| Personality Disorders (Conditional RP^2^P = 0.61) | | | | | | |
| Intercept | -0.66 | -1.64, 0.32 | 0.50 | 69.20 | -1.327 | 0.189 |
| Time | -0.02 | -0.11, 0.06 | 0.04 | 97.19 | -0.547 | 0.586 |
| Age | 0.00 | -0.01, 0.02 | 0.01 | 68.58 | 0.519 | 0.606 |
| Sex | 0.46 | 0.04, 0.88 | 0.21 | 65.96 | 2.139 | 0.036 |
| β: Standardized beta; diagnostic categories are related to DSM-IV. | | | | | | |

**Supplementary Table 7.** Effect sizes for the association between DSM-5 symptoms and functional impairment across WHODAS II domains during 18 months in outpatients and daycare patients (*N* = 155)

|  | β | 95% CI |
| --- | --- | --- |
| Social Anxiety Disorder | 0.69 | 0.48, 0.90 |
| Fear of acting in a way or showing anxiety symptoms that will be negatively evaluated (B) | 0.69 | 0.48, 0.90 |
| Conversion Disorder | 0.58 | 0.29, 0.87 |
| Symptoms of altered voluntary motor or sensory function (A) | 0.58 | 0.29, 0.87 |
| Dissociative Identity Disorder | 0.50 | 0.23, 0.78 |
| Disruption of identity (A) | 0.56 | 0.29, 0.83 |
| Recurrent gaps in the recall of everyday events, important personal information, and/or traumatic events (B) | 0.44 | 0.17, 0.72 |
| Major Depression | 0.48 | 0.28, 0.69 |
| Fatigue or loss of energy (A6) | 0.68 | 0.47, 0.88 |
| Diminished ability to think or concentrate, or indecisiveness (A8) | 0.66 | 0.46, 0.85 |
| Depressed mood (A1) | 0.62 | 0.42, 0.82 |
| Markedly diminished interest or pleasure (A2) | 0.60 | 0.39, 0.80 |
| Feelings of worthlessness or excessive or inappropriate guilt (A7) | 0.59 | 0.36, 0.82 |
| Psychomotor agitation or retardation (A5) | 0.49 | 0.29, 0.68 |
| Insomnia or hypersomnia (A4) | 0.32 | 0.12, 0.52 |
| Recurrent thoughts of death, suicidal ideation, or a suicide attempt or a specific plan for committing suicide (A9) | 0.23 | 0.04, 0.42 |
| Significant weight loss when not dieting or weight gain (A3) | 0.18 | -0.06, 0.41 |
| Depersonalization-, Derealization Disorder | 0.48 | 0.22, 0.73 |
| Depersonalization (A1) | 0.51 | 0.24, 0.77 |
| Derealization (A2) | 0.45 | 0.20, 0.70 |
| Posttraumatic Stress Disorder | 0.38 | 0.20, 0.56 |
| Dissociative reactions (e.g., flashbacks) (B3) | 0.52 | 0.31, 0.73 |
| Markedly diminished interest or participation in significant activities (D5) | 0.48 | 0.29, 0.68 |
| Intense or prolonged psychological distress at exposure to internal or external cues (B4) | 0.47 | 0.29, 0.66 |
| Problems with concentration (E5) | 0.43 | 0.27, 0.59 |
| Irritable behavior and angry outbursts (E1) | 0.40 | 0.14, 0.66 |
| Avoidance of or efforts to avoid external reminders (C2) | 0.37 | 0.21, 0.53 |
| Marked physiological reactions to internal or external cues (B5) | 0.35 | 0.19, 0.52 |
| Recurrent, involuntary, and intrusive distressing memories of the traumatic event(s) (B1) | 0.35 | 0.18, 0.52 |
| Feelings of detachment or estrangement from others (D6) | 0.34 | 0.14, 0.54 |
| Recurrent distressing dreams with content and/or affect related to the traumatic event(s) (B2) | 0.32 | 0.16, 0.49 |
| Sleep disturbance (E6) | 0.32 | 0.12, 0.52 |
| Exaggerated startle response (E4) | 0.32 | 0.15, 0.48 |
| Persistent and exaggerated negative beliefs or expectations (D2) | 0.28 | 0.11, 0.45 |
| Specific Phobia | 0.38 | 0.20, 0.56 |
| A phobic object or situation is actively avoided or endured with intense fear or anxiety (C) | 0.37 | 0.21, 0.53 |
| Generalized Anxiety Disorder | 0.38 | 0.20, 0.56 |
| Restlessness or feeling keyed up or on edge (C1) | 0.49 | 0.29, 0.68 |
| Difficulty concentrating or mind going blank (C3) | 0.44 | 0.26, 0.63 |
| Sleep disturbance (C6) | 0.32 | 0.12, 0.52 |
| Being easily fatigued (C2) | 0.30 | 0.14, 0.46 |
| Irritability (C4) | 0.23 | 0.02, 0.45 |

| Agoraphobia | 0.38 | 0.200, 0.56 |
| --- | --- | --- |
| Fear of using public transportation (A1) | 0.40 | 0.23, 0.56 |
| Fearing or avoiding situations (B) | 0.37 | 0.21, 0.53 |
| Fear of being in open spaces (A2) | 0.35 | 0.19, 0.52 |
| Fear of being outside of the home alone (A5) | 0.32 | 0.15, 0.49 |
| Fear of standing in line or being in a crowd (A4) | 0.32 | 0.16, 0.49 |
| Panic Disorder | 0.29 | 0.11, 0.46 |
| Derealization or depersonalization (A11) | 0.48 | 0.23, 0.73 |
| Sensations of shortness of breath or smothering (A4) | 0.42 | 0.26, 0.59 |
| Fear of losing control or 'going crazy' (A12) | 0.34 | 0.18, 0.50 |
| Recurrent unexpected panic attacks (A) | 0.31 | 0.15, 0.48 |
| Trembling or shaking (A3) | 0.29 | 0.12, 0.45 |
| Chest pain or discomfort (A6) | 0.23 | 0.07, 0.40 |
| Paresthesias (numbness or tingling sensations) (A10) | 0.23 | 0.06, 0.40 |
| Chills or heat sensations (A9) | 0.23 | 0.06, 0.39 |
| Nausea or abdominal distress (A7) | 0.18 | 0.02, 0.35 |
| Feeling dizzy, unsteady, light-headed, or faint (A8) | 0.15 | -0.01, 0.32 |
| Dissociative Amnesia | 0.22 | -0.05, 0.48 |
| Inability to recall important autobiographical information, usually of a traumatic or stressful nature (A) | 0.22 | -0.05, 0.48 |
| Obsessive-Compulsive Disorder | 0.21 | 0.04, 0.38 |
| Repetitive behaviors in response to an obsession or according to rules that must be applied rigidly (A2.1) | 0.21 | 0.04, 0.38 |
| β: Standardized beta;P Pletters in parenthesis represent the DSM-5 criterion. | | |

**Supplementary Table 8.** Effect sizes for the association between DSM-5 symptoms and functional impairment in WHODAS II domain 'Understanding and Communication' during 18 months in outpatients and daycare patients (*N* = 155)

|  | β | 95% CI |
| --- | --- | --- |
| Conversion Disorder | 0.73 | 0.43, 1.04 |
| Symptoms of altered voluntary motor or sensory function (A) | 0.73 | 0.43, 1.04 |
| Social Anxiety Disorder | 0.73 | 0.50, 0.95 |
| Fear of acting in a way or showing anxiety symptoms that will be negatively evaluated (B) | 0.73 | 0.50, 0.95 |
| Dissociative Identity Disorder | 0.71 | 0.43, 0.99 |
| Disruption of identity (A) | 0.74 | 0.46, 1.02 |
| Recurrent gaps in the recall of everyday events, important personal information, and/or traumatic events (B) | 0.68 | 0.40, 0.96 |
| Depersonalization-, Derealization Disorder | 0.67 | 0.41, 0.94 |
| Depersonalization (A1) | 0.70 | 0.43, 0.98 |
| Derealization (A2) | 0.65 | 0.39, 0.90 |
| Major Depression | 0.52 | 0.30, 0.74 |
| Diminished ability to think or concentrate, or indecisiveness (A8) | 0.78 | 0.58, 0.98 |
| Fatigue or loss of energy (A6) | 0.71 | 0.50, 0.93 |
| Depressed mood (A1) | 0.70 | 0.48, 0.91 |
| Markedly diminished interest or pleasure (A2) | 0.65 | 0.43, 0.87 |
| Feelings of worthlessness or excessive or inappropriate guilt (A7) | 0.64 | 0.39, 0.88 |
| Psychomotor agitation or retardation (A5) | 0.50 | 0.29, 0.71 |
| Recurrent thoughts of death, suicidal ideation, or a suicide attempt or a specific plan for committing suicide (A9) | 0.32 | 0.12, 0.52 |
| Insomnia or hypersomnia (A4) | 0.23 | 0.02, 0.45 |
| Significant weight loss when not dieting or weight gain (A3) | 0.18 | -0.08, 0.44 |
| Posttraumatic Stress Disorder | 0.42 | 0.22, 0.61 |
| Dissociative reactions (e.g., flashbacks) (B3) | 0.59 | 0.37, 0.81 |
| Problems with concentration (E5) | 0.57 | 0.40, 0.74 |
| Intense or prolonged psychological distress at exposure to internal or external cues (B4) | 0.55 | 0.36, 0.75 |
| Markedly diminished interest or participation in significant activities (D5) | 0.53 | 0.33, 0.74 |
| Recurrent, involuntary, and intrusive distressing memories of the traumatic event(s) (B1) | 0.47 | 0.30, 0.64 |
| Irritable behavior and angry outbursts (E1) | 0.46 | 0.19, 0.73 |
| Marked physiological reactions to internal or external cues (B5) | 0.40 | 0.22, 0.58 |
| Feelings of detachment or estrangement from others (D6) | 0.39 | 0.18, 0.61 |
| Avoidance of or efforts to avoid external reminders (C2) | 0.35 | 0.17, 0.53 |
| Recurrent distressing dreams with content and/or affect related to the traumatic event(s) (B2) | 0.29 | 0.11, 0.47 |
| Exaggerated startle response (E4) | 0.28 | 0.10, 0.46 |
| Persistent and exaggerated negative beliefs or expectations (D2) | 0.27 | 0.09, 0.45 |
| Sleep disturbance (E6) | 0.23 | 0.02, 0.45 |
| Generalized Anxiety Disorder | 0.39 | 0.18, 0.59 |
| Difficulty concentrating or mind going blank (C3) | 0.62 | 0.43, 0.8 |
| Restlessness or feeling keyed up or on edge (C1) | 0.50 | 0.29, 0.71 |
| Being easily fatigued (C2) | 0.30 | 0.12, 0.48 |
| Irritability (C4) | 0.28 | 0.06, 0.51 |
| Sleep disturbance (C6) | 0.23 | 0.02, 0.45 |
| Dissociative Amnesia | 0.38 | 0.10, 0.65 |
| Inability to recall important autobiographical information, usually of a traumatic or stressful nature (A) | 0.38 | 0.10, 0.65 |
| Specific Phobia | 0.35 | 0.17, 0.53 |
| A phobic object or situation is actively avoided or endured with intense fear or anxiety (C) | 0.35 | 0.17, 0.53 |
| Agoraphobia | 0.33 | 0.14, 0.51 |
| Fear of being outside of the home alone (A5) | 0.40 | 0.22, 0.58 |
| Fearing or avoiding situations (B) | 0.35 | 0.17, 0.53 |
| Fear of using public transportation (A1) | 0.34 | 0.15, 0.52 |
| Fear of standing in line or being in a crowd (A4) | 0.29 | 0.11, 0.47 |
| Fear of being in open spaces (A2) | 0.26 | 0.08, 0.45 |
| Panic Disorder | 0.32 | 0.13, 0.51 |
| Derealization or depersonalization (A11) | 0.68 | 0.42, 0.94 |
| Fear of losing control or 'going crazy' (A12) | 0.46 | 0.29, 0.63 |
| Sensations of shortness of breath or smothering (A4) | 0.46 | 0.28, 0.64 |
| Trembling or shaking (A3) | 0.29 | 0.12, 0.47 |
| Chills or heat sensations (A9) | 0.27 | 0.09, 0.45 |
| Chest pain or discomfort (A6) | 0.24 | 0.07, 0.42 |
| Nausea or abdominal distress (A7) | 0.23 | 0.05, 0.41 |
| Paresthesias (numbness or tingling sensations) (PD A10) | 0.20 | 0.02, 0.38 |
| Recurrent unexpected panic attacks (A) | 0.18 | 0.00, 0.37 |
| Feeling dizzy, unsteady, light-headed, or faint (A8) | 0.17 | -0.01, 0.35 |
| Obsessive-Compulsive Disorder | 0.27 | 0.09, 0.45 |
| Repetitive behaviors in response to an obsession or according to rules that must be applied rigidly (A2.1) | 0.27 | 0.09, 0.45 |
| β: Standardized beta;P Pletters in parenthesis represent the DSM-5 criterion. | | |

**Supplementary Table 9.** Effect sizes for the association between DSM-5 symptoms and functional impairment in WHODAS II domain 'Getting Around' during 18 months in outpatients and daycare patients (*N* = 155)

|  | β | 95% CI |
| --- | --- | --- |
| Conversion Disorder | 0.32 | -0.01, 0.65 |
| Symptoms of altered voluntary motor or sensory function (A) | 0.32 | -0.01, 0.65 |
| Agoraphobia | 0.23 | 0.05, 0.42 |
| Fear of using public transportation (A1) | 0.38 | 0.19, 0.57 |
| Fear of being in open spaces (A2) | 0.35 | 0.17, 0.54 |
| Fearing or avoiding situations (B) | 0.23 | 0.04, 0.41 |
| Fear of standing in line or being in a crowd (A4) | 0.17 | -0.02, 0.36 |
| Fear of being outside of the home alone (A5) | 0.04 | -0.15, 0.24 |
| Specific Phobia | 0.23 | 0.04, 0.41 |
| A phobic object or situation is actively avoided or endured with intense fear or anxiety (C) | 0.23 | 0.04, 0.41 |
| Social Anxiety Disorder | 0.21 | -0.05, 0.47 |
| Fear of acting in a way or showing anxiety symptoms that will be negatively evaluated (B) | 0.21 | -0.05, 0.47 |
| Major Depression | 0.20 | -0.04, 0.43 |
| Diminished ability to think or concentrate, or indecisiveness (A8) | 0.27 | 0.04, 0.51 |
| Feelings of worthlessness or excessive or inappropriate guilt (A7) | 0.24 | -0.03, 0.50 |
| Markedly diminished interest or pleasure (A2) | 0.22 | -0.02, 0.47 |
| Fatigue or loss of energy (A6) | 0.22 | -0.03, 0.47 |
| Depressed mood (A1) | 0.22 | -0.02, 0.46 |
| Psychomotor agitation or retardation (A5) | 0.22 | -0.01, 0.44 |
| Significant weight loss when not dieting or weight gain (A3) | 0.18 | -0.08, 0.44 |
| Insomnia or hypersomnia (A4) | 0.15 | -0.07, 0.37 |
| Recurrent thoughts of death, suicidal ideation, or a suicide attempt or a specific plan for committing suicide (A9) | 0.04 | -0.17, 0.25 |
| Panic Disorder | 0.16 | -0.04, 0.36 |
| Paresthesias (numbness or tingling sensations) (A10) | 0.27 | 0.08, 0.45 |
| Sensations of shortness of breath or smothering (A4) | 0.24 | 0.05, 0.43 |
| Nausea or abdominal distress (A7) | 0.23 | 0.05, 0.41 |
| Chest pain or discomfort (A6) | 0.17 | -0.01, 0.35 |
| Recurrent unexpected panic attacks (A) | 0.17 | -0.02, 0.36 |
| Trembling or shaking (A3) | 0.14 | -0.05, 0.32 |
| Chills or heat sensations (A9) | 0.13 | -0.05, 0.32 |
| Fear of losing control or 'going crazy' (A12) | 0.10 | -0.09, 0.29 |
| Derealization or depersonalization (A11) | 0.09 | -0.19, 0.38 |
| Feeling dizzy, unsteady, light-headed, or faint (A8) | 0.06 | -0.13, 0.24 |
| Posttraumatic Stress Disorder | 0.16 | -0.05, 0.37 |
| Dissociative reactions (e.g., flashbacks) (B3) | 0.33 | 0.09, 0.57 |
| Marked physiological reactions to internal or external cues (B5) | 0.28 | 0.09, 0.46 |
| Intense or prolonged psychological distress at exposure to internal or external cues (B4) | 0.25 | 0.03, 0.47 |
| Avoidance of or efforts to avoid external reminders (C2) | 0.23 | 0.04, 0.41 |
| Recurrent distressing dreams with content and/or affect related to the traumatic event(s) (B2) | 0.19 | 0.00, 0.38 |
| Exaggerated startle response (E4) | 0.18 | 0.00, 0.37 |
| Markedly diminished interest or participation in significant activities (D5) | 0.18 | -0.05, 0.40 |
| Sleep disturbance (E6) | 0.15 | -0.07, 0.37 |
| Problems with concentration (E5) | 0.14 | -0.05, 0.33 |
| Recurrent, involuntary, and intrusive distressing memories of the traumatic event(s) (B1) | 0.10 | -0.09, 0.29 |
| Persistent and exaggerated negative beliefs or expectations (D2) | 0.08 | -0.11, 0.26 |
| Irritable behavior and angry outbursts (E1) | 0.05 | -0.23, 0.33 |
| Feelings of detachment or estrangement from others (D6) | -0.02 | -0.24, 0.21 |
| Generalized Anxiety Disorder | 0.12 | -0.09, 0.34 |
| Restlessness or feeling keyed up or on edge (C1) | 0.22 | -0.01, 0.44 |
| Sleep disturbance (C6) | 0.15 | -0.07, 0.37 |
| Difficulty concentrating or mind going blank (C3) | 0.12 | -0.09, 0.34 |
| Being easily fatigued (C2) | 0.10 | -0.08, 0.29 |
| Irritability (C4) | 0.01 | -0.22, 0.24 |
| Dissociative Identity Disorder | 0.10 | -0.21, 0.41 |
| Disruption of identity (A) | 0.14 | -0.18, 0.45 |
| Recurrent gaps in the recall of everyday events, important personal information, and/or traumatic events (B) | 0.06 | -0.24, 0.37 |
| Obsessive-Compulsive Disorder | 0.10 | -0.10, 0.29 |
| Repetitive behaviors in response to an obsession or according to rules that must be applied rigidly (A2.1) | 0.10 | -0.10, 0.29 |
| Depersonalization-, Derealization Disorder | 0.08 | -0.22, 0.37 |
| Depersonalization (A1) | 0.08 | -0.22, 0.38 |
| Derealization (A2) | 0.07 | -0.21, 0.36 |
| Dissociative Amnesia | 0.07 | -0.21, 0.36 |
| Inability to recall important autobiographical information, usually of a traumatic or stressful nature (A) | 0.07 | -0.21, 0.36 |
| β: Standardized beta; letters in parenthesis represent the DSM-5 criterion. | | |

**Supplementary Table 10.** Effect sizes for the association between DSM-5 symptoms and functional impairment in WHODAS II domain 'Self-Care' during 18 months in outpatients and daycare patients (*N* = 155)

|  | β | 95% CI |
| --- | --- | --- |
| Conversion Disorder | 0.56 | 0.24, 0.87 |
| Symptoms of altered voluntary motor or sensory function (A) | 0.56 | 0.24, 0.87 |
| Social Anxiety Disorder | 0.52 | 0.28, 0.76 |
| Fear of acting in a way or showing anxiety symptoms that will be negatively evaluated (B) | 0.52 | 0.28, 0.76 |
| Dissociative Identity Disorder | 0.44 | 0.14, 0.74 |
| Disruption of identity (A) | 0.51 | 0.21, 0.80 |
| Recurrent gaps in the recall of everyday events, important personal information, and/or traumatic events (B) | 0.37 | 0.08, 0.67 |
| Depersonalization-, Derealization Disorder | 0.39 | 0.11, 0.68 |
| Depersonalization (A1) | 0.43 | 0.14, 0.73 |
| Derealization (A2) | 0.35 | 0.08, 0.63 |
| Major Depression | 0.31 | 0.07, 0.55 |
| Diminished ability to think or concentrate, or indecisiveness (A8) | 0.43 | 0.20, 0.66 |
| Depressed mood (A1) | 0.43 | 0.20, 0.66 |
| Fatigue or loss of energy (A6) | 0.40 | 0.16, 0.64 |
| Markedly diminished interest or pleasure (A2) | 0.39 | 0.15, 0.63 |
| Feelings of worthlessness or excessive or inappropriate guilt (A7) | 0.33 | 0.07, 0.59 |
| Psychomotor agitation or retardation (A5) | 0.25 | 0.03, 0.47 |
| Significant weight loss when not dieting or weight gain (A3) | 0.23 | -0.05, 0.50 |
| Recurrent thoughts of death, suicidal ideation, or a suicide attempt or a specific plan for committing suicide (A9) | 0.21 | 0.01, 0.41 |
| Insomnia or hypersomnia (A4) | 0.11 | -0.11, 0.33 |
| Posttraumatic Stress Disorder | 0.30 | 0.09, 0.50 |
| Dissociative reactions (e.g., flashbacks) (B3) | 0.49 | 0.25, 0.72 |
| Irritable behavior and angry outbursts (E1) | 0.46 | 0.19, 0.74 |
| Intense or prolonged psychological distress at exposure to internal or external cues (B4) | 0.42 | 0.21, 0.62 |
| Markedly diminished interest or participation in significant activities (D5) | 0.37 | 0.16, 0.59 |
| Marked physiological reactions to internal or external cues (B5) | 0.33 | 0.15, 0.51 |
| Problems with concentration (E5) | 0.31 | 0.12, 0.49 |
| Avoidance of or efforts to avoid external reminders (C2) | 0.30 | 0.12, 0.48 |
| Recurrent, involuntary, and intrusive distressing memories of the traumatic event(s) (B1) | 0.27 | 0.09, 0.46 |
| Recurrent distressing dreams with content and/or affect related to the traumatic event(s) (B2) | 0.24 | 0.06, 0.42 |
| Exaggerated startle response (E4) | 0.22 | 0.03, 0.40 |
| Persistent and exaggerated negative beliefs or expectations (D2) | 0.21 | 0.02, 0.39 |
| Feelings of detachment or estrangement from others (D6) | 0.16 | -0.06, 0.38 |
| Sleep disturbance (E6) | 0.11 | -0.11, 0.33 |
| Specific Phobia | 0.30 | 0.12, 0.48 |
| A phobic object or situation is actively avoided or endured with intense fear or anxiety (C) | 0.30 | 0.12, 0.48 |
| Agoraphobia | 0.24 | 0.05, 0.42 |
| Fear of being outside of the home alone (A5) | 0.31 | 0.13, 0.50 |
| Fearing or avoiding situations (B) | 0.30 | 0.12, 0.48 |
| Fear of being in open spaces (A2) | 0.27 | 0.08, 0.45 |
| Fear of standing in line or being in a crowd (A4) | 0.18 | 0.00, 0.37 |
| Fear of using public transportation (A1) | 0.12 | -0.07, 0.32 |
| Panic Disorder | 0.23 | 0.04, 0.42 |
| Sensations of shortness of breath or smothering (A4) | 0.39 | 0.21, 0.58 |
| Derealization or depersonalization (A11) | 0.38 | 0.1, 0.65 |
| Fear of losing control or 'going crazy' (A12) | 0.30 | 0.12, 0.48 |
| Recurrent unexpected panic attacks (A) | 0.30 | 0.12, 0.49 |
| Paresthesias (numbness or tingling sensations) (A10) | 0.21 | 0.03, 0.40 |
| Chest pain or discomfort (A6) | 0.20 | 0.02, 0.38 |
| Nausea or abdominal distress (A7) | 0.20 | 0.02, 0.38 |
| Chills or heat sensations (A9) | 0.13 | -0.05, 0.32 |
| Trembling or shaking (A3) | 0.10 | -0.08, 0.29 |
| Feeling dizzy, unsteady, light-headed, or faint (A8) | 0.08 | -0.11, 0.27 |
| Generalized Anxiety Disorder | 0.20 | -0.01, 0.42 |
| Difficulty concentrating or mind going blank (C3) | 0.28 | 0.08, 0.49 |
| Restlessness or feeling keyed up or on edge (C1) | 0.25 | 0.03, 0.47 |
| Irritability (C4) | 0.25 | 0.02, 0.48 |
| Being easily fatigued (C2) | 0.12 | -0.06, 0.30 |
| Sleep disturbance (C6) | 0.11 | -0.11, 0.33 |
| Dissociative Amnesia | 0.14 | -0.14, 0.43 |
| Inability to recall important autobiographical information, usually of a traumatic or stressful nature (A) | 0.14 | -0.14, 0.43 |
| Obsessive-Compulsive Disorder | 0.11 | -0.08, 0.30 |
| Repetitive behaviors in response to an obsession or according to rules that must be applied rigidly (A2.1) | 0.11 | -0.08, 0.30 |
| β: Standardized beta;P Pletters in parenthesis represent the DSM-5 criterion. | | |

**Supplementary Table 11.** Effect sizes for the association between DSM-5 symptoms and functional impairment in WHODAS II domain 'Getting Along with People' during 18 months in outpatients and daycare patients (*N* = 155)

|  | β | 95% CI |
| --- | --- | --- |
| Social Anxiety Disorder | 0.62 | 0.40, 0.83 |
| Fear of acting in a way or showing anxiety symptoms that will be negatively evaluated (B) | 0.62 | 0.40, 0.83 |
| Depersonalization-, Derealization Disorder | 0.44 | 0.18, 0.69 |
| Depersonalization (A1) | 0.48 | 0.22, 0.74 |
| Derealization (A2) | 0.40 | 0.15, 0.65 |
| Major Depression | 0.36 | 0.15, 0.57 |
| Markedly diminished interest or pleasure (A2) | 0.55 | 0.34, 0.75 |
| Feelings of worthlessness or excessive or inappropriate guilt (A7) | 0.52 | 0.29, 0.75 |
| Fatigue or loss of energy (A6) | 0.52 | 0.31, 0.73 |
| Diminished ability to think or concentrate, or indecisiveness (A8) | 0.52 | 0.31, 0.72 |
| Depressed mood (A1) | 0.48 | 0.27, 0.69 |
| Psychomotor agitation or retardation (A5) | 0.39 | 0.19, 0.58 |
| Insomnia or hypersomnia (A4) | 0.21 | 0.01, 0.41 |
| Recurrent thoughts of death, suicidal ideation, or a suicide attempt or a specific plan for committing suicide (A9) | 0.14 | -0.05, 0.33 |
| Significant weight loss when not dieting or weight gain (A3) | -0.07 | -0.31, 0.17 |
| Conversion Disorder | 0.35 | 0.05, 0.65 |
| Symptoms of altered voluntary motor or sensory function (A) | 0.35 | 0.05, 0.65 |
| Dissociative Identity Disorder | 0.35 | 0.07, 0.62 |
| Disruption of identity (A) | 0.42 | 0.15, 0.7 |
| Recurrent gaps in the recall of everyday events, important personal information, and/or traumatic events (B) | 0.27 | 0.00, 0.55 |
| Specific Phobia | 0.29 | 0.13, 0.46 |
| A phobic object or situation is actively avoided or endured with intense fear or anxiety (C) | 0.29 | 0.13, 0.46 |
| Generalized Anxiety Disorder | 0.28 | 0.09, 0.47 |
| Restlessness or feeling keyed up or on edge (C1) | 0.39 | 0.19, 0.58 |
| Difficulty concentrating or mind going blank (C3) | 0.35 | 0.16, 0.54 |
| Being easily fatigued (C2) | 0.27 | 0.10, 0.43 |
| Sleep disturbance (C6) | 0.21 | 0.01, 0.41 |
| Irritability (C4) | 0.17 | -0.04, 0.38 |
| Posttraumatic Stress Disorder | 0.28 | 0.10, 0.47 |
| Feelings of detachment or estrangement from others (D6) | 0.49 | 0.29, 0.68 |
| Markedly diminished interest or participation in significant activities (D5) | 0.47 | 0.28, 0.66 |
| Problems with concentration (E5) | 0.31 | 0.14, 0.47 |
| Avoidance of or efforts to avoid external reminders (C2) | 0.29 | 0.13, 0.46 |
| Recurrent, involuntary, and intrusive distressing memories of the traumatic event(s) (B1) | 0.29 | 0.12, 0.46 |
| Dissociative reactions (e.g., flashbacks) (B3) | 0.28 | 0.06, 0.49 |
| Irritable behavior and angry outbursts (E1) | 0.27 | 0.01, 0.53 |
| Persistent and exaggerated negative beliefs or expectations (D2) | 0.26 | 0.10, 0.43 |
| Intense or prolonged psychological distress at exposure to internal or external cues (B4) | 0.25 | 0.05, 0.45 |
| Recurrent distressing dreams with content and/or affect related to the traumatic event(s) (B2) | 0.22 | 0.05, 0.39 |
| Sleep disturbance (E6) | 0.21 | 0.01, 0.41 |
| Exaggerated startle response (E4) | 0.18 | 0.02, 0.35 |
| Marked physiological reactions to internal or external cues (B5) | 0.17 | 0.00, 0.34 |
| Agoraphobia | 0.24 | 0.07, 0.41 |
| Fearing or avoiding situations (B) | 0.29 | 0.13, 0.46 |
| Fear of using public transportation (A1) | 0.28 | 0.11, 0.45 |
| Fear of standing in line or being in a crowd (A4) | 0.27 | 0.10, 0.43 |
| Fear of being outside of the home alone (A5) | 0.20 | 0.03, 0.37 |
| Fear of being in open spaces (A2) | 0.18 | 0.01, 0.35 |
| Dissociative Amnesia | 0.22 | -0.04, 0.48 |
| Inability to recall important autobiographical information, usually of a traumatic or stressful nature (A) | 0.22 | -0.04, 0.48 |
| Panic Disorder | 0.21 | 0.04, 0.39 |
| Derealization or depersonalization (A11) | 0.43 | 0.18, 0.68 |
| Fear of losing control or 'going crazy' (A12) | 0.29 | 0.13, 0.46 |
| Sensations of shortness of breath or smothering (A4) | 0.29 | 0.12, 0.46 |
| Trembling or shaking (A3) | 0.23 | 0.06, 0.39 |
| Recurrent unexpected panic attacks (A) | 0.20 | 0.03, 0.37 |
| Chills or heat sensations (A9) | 0.18 | 0.01, 0.35 |
| Feeling dizzy, unsteady, light-headed, or faint (A8) | 0.18 | 0.01, 0.35 |
| Paresthesias (numbness or tingling sensations) (A10) | 0.15 | -0.03, 0.32 |
| Nausea or abdominal distress (A7) | 0.10 | -0.06, 0.27 |
| Chest pain or discomfort (A6) | 0.10 | -0.06, 0.27 |
| Obsessive-Compulsive Disorder | 0.17 | 0.00, 0.34 |
| Repetitive behaviors in response to an obsession or according to rules that must be applied rigidly (A2.1) | 0.17 | 0.00, 0.34 |
| β: Standardized beta;P Pletters in parenthesis represent the DSM-5 criterion. | | |

**Supplementary Table 12.** Effect sizes for the association between DSM-5 symptoms and functional impairment in WHODAS II domain 'Life Activities' during 18 months in outpatients and daycare patients (*N* = 155)

|  | β | 95% CI |
| --- | --- | --- |
| Social Anxiety Disorder | 0.54 | 0.29, 0.79 |
| Fear of acting in a way or showing anxiety symptoms that will be negatively evaluated (B) | 0.54 | 0.29, 0.79 |
| Conversion Disorder | 0.45 | 0.11, 0.78 |
| Symptoms of altered voluntary motor or sensory function (A) | 0.45 | 0.11, 0.78 |
| Major Depression | 0.41 | 0.17, 0.64 |
| Fatigue or loss of energy (A6) | 0.76 | 0.53, 0.99 |
| Diminished ability to think or concentrate, or indecisiveness (A8) | 0.51 | 0.27, 0.75 |
| Markedly diminished interest or pleasure (A2) | 0.47 | 0.22, 0.71 |
| Depressed mood (A1) | 0.43 | 0.19, 0.67 |
| Psychomotor agitation or retardation (A5) | 0.40 | 0.17, 0.62 |
| Feelings of worthlessness or excessive or inappropriate guilt (A7) | 0.38 | 0.11, 0.65 |
| Insomnia or hypersomnia (A4) | 0.36 | 0.14, 0.58 |
| Significant weight loss when not dieting or weight gain (A3) | 0.26 | -0.01, 0.52 |
| Recurrent thoughts of death, suicidal ideation, or a suicide attempt or a specific plan for committing suicide (A9) | 0.09 | -0.12, 0.31 |
| Dissociative Identity Disorder | 0.37 | 0.05, 0.68 |
| Disruption of identity (A) | 0.41 | 0.09, 0.72 |
| Recurrent gaps in the recall of everyday events, important personal information, and/or traumatic events (B) | 0.33 | 0.02, 0.65 |
| Generalized Anxiety Disorder | 0.32 | 0.10, 0.54 |
| Restlessness or feeling keyed up or on edge (C1) | 0.40 | 0.17, 0.62 |
| Sleep disturbance (C6) | 0.36 | 0.14, 0.58 |
| Being easily fatigued (C2) | 0.33 | 0.14, 0.51 |
| Difficulty concentrating or mind going blank (C3) | 0.31 | 0.09, 0.52 |
| Irritability (C4) | 0.21 | -0.03, 0.45 |
| Posttraumatic Stress Disorder | 0.30 | 0.09, 0.51 |
| Dissociative reactions (e.g., flashbacks) (B3) | 0.40 | 0.15, 0.64 |
| Markedly diminished interest or participation in significant activities (D5) | 0.40 | 0.17, 0.62 |
| Irritable behavior and angry outbursts (E1) | 0.38 | 0.09, 0.68 |
| Intense or prolonged psychological distress at exposure to internal or external cues (B4) | 0.37 | 0.15, 0.58 |
| Sleep disturbance (E6) | 0.36 | 0.14, 0.58 |
| Problems with concentration (E5) | 0.35 | 0.16, 0.54 |
| Marked physiological reactions to internal or external cues (B5) | 0.27 | 0.08, 0.46 |
| Exaggerated startle response (E4) | 0.26 | 0.07, 0.45 |
| Recurrent distressing dreams with content and/or affect related to the traumatic event(s) (B2) | 0.26 | 0.07, 0.45 |
| Persistent and exaggerated negative beliefs or expectations (D2) | 0.23 | 0.03, 0.42 |
| Recurrent, involuntary, and intrusive distressing memories of the traumatic event(s) (B1) | 0.22 | 0.03, 0.41 |
| Feelings of detachment or estrangement from others (D6) | 0.21 | -0.02, 0.44 |
| Avoidance of or efforts to avoid external reminders (C2) | 0.21 | 0.02, 0.40 |
| Depersonalization-, Derealization Disorder | 0.27 | -0.03, 0.57 |
| Depersonalization (A1) | 0.28 | -0.03, 0.58 |
| Derealization (A2) | 0.26 | -0.03, 0.55 |
| Agoraphobia | 0.24 | 0.05, 0.44 |
| Fear of being in open spaces (A2) | 0.35 | 0.16, 0.54 |
| Fear of standing in line or being in a crowd (A4) | 0.28 | 0.09, 0.47 |
| Fearing or avoiding situations (B) | 0.21 | 0.02, 0.4 |
| Fear of using public transportation (A1) | 0.20 | 0.00, 0.40 |
| Fear of being outside of the home alone (A5) | 0.18 | -0.01, 0.38 |
| Specific Phobia | 0.21 | 0.02, 0.4 |
| A phobic object or situation is actively avoided or endured with intense fear or anxiety (C) | 0.21 | 0.02, 0.4 |
| Panic Disorder | 0.17 | -0.03, 0.37 |
| Recurrent unexpected panic attacks (A) | 0.30 | 0.10, 0.49 |
| Derealization or depersonalization (A11) | 0.25 | -0.04, 0.54 |
| Trembling or shaking (A3) | 0.24 | 0.05, 0.43 |
| Fear of losing control or 'going crazy' (A12) | 0.23 | 0.04, 0.42 |
| Sensations of shortness of breath or smothering (A4) | 0.22 | 0.02, 0.42 |
| Chest pain or discomfort (A6) | 0.10 | -0.09, 0.29 |
| Nausea or abdominal distress (A7) | 0.09 | -0.1, 0.28 |
| Paresthesias (numbness or tingling sensations) (A10) | 0.09 | -0.11, 0.28 |
| Chills or heat sensations (A9) | 0.08 | -0.11, 0.27 |
| Feeling dizzy, unsteady, light-headed, or faint (A8) | 0.07 | -0.12, 0.27 |
| Dissociative Amnesia | 0.12 | -0.17, 0.42 |
| Inability to recall important autobiographical information, usually of a traumatic or stressful nature (A) | 0.12 | -0.17, 0.42 |
| Obsessive-Compulsive Disorder | 0.07 | -0.12, 0.27 |
| Repetitive behaviors in response to an obsession or according to rules that must be applied rigidly (A2.1) | 0.07 | -0.12, 0.27 |
| β: Standardized beta;P Pletters in parenthesis represent the DSM-5 criterion. | | |

**Supplementary Table 13.** Effect sizes for the association between DSM-5 symptoms and functional impairment in WHODAS II domain 'Participation in Society' during 18 months in outpatients and daycare patients (*N* = 155)

|  | β | 95% CI |
| --- | --- | --- |
| Social Anxiety Disorder | 0.66 | 0.43, 0.89 |
| Fear of acting in a way or showing anxiety symptoms that will be negatively evaluated (B) | 0.66 | 0.43, 0.89 |
| Major Depression | 0.48 | 0.26, 0.70 |
| Depressed mood (A1) | 0.65 | 0.43, 0.86 |
| Feelings of worthlessness or excessive or inappropriate guilt (A7) | 0.62 | 0.38, 0.86 |
| Markedly diminished interest or pleasure (A2) | 0.59 | 0.37, 0.81 |
| Diminished ability to think or concentrate, or indecisiveness (A8) | 0.59 | 0.37, 0.80 |
| Fatigue or loss of energy (A6) | 0.55 | 0.32, 0.78 |
| Psychomotor agitation or retardation (A5) | 0.50 | 0.29, 0.71 |
| Insomnia or hypersomnia (A4) | 0.35 | 0.14, 0.56 |
| Recurrent thoughts of death, suicidal ideation, or a suicide attempt or a specific plan for committing suicide (A9) | 0.28 | 0.09, 0.48 |
| Significant weight loss when not dieting or weight gain (A3) | 0.19 | -0.04, 0.42 |
| Depersonalization-, Derealization Disorder | 0.40 | 0.12, 0.67 |
| Depersonalization (A1) | 0.43 | 0.14, 0.71 |
| Derealization (A2) | 0.37 | 0.10, 0.64 |
| Dissociative Identity Disorder | 0.40 | 0.11, 0.70 |
| Disruption of identity (A) | 0.47 | 0.18, 0.77 |
| Recurrent gaps in the recall of everyday events, important personal information, and/or traumatic events (B) | 0.34 | 0.04, 0.63 |
| Specific Phobia | 0.39 | 0.22, 0.56 |
| A phobic object or situation is actively avoided or endured with intense fear or anxiety (C) | 0.39 | 0.22, 0.56 |
| Posttraumatic Stress Disorder | 0.37 | 0.17, 0.56 |
| Dissociative reactions (e.g., flashbacks) (B3) | 0.48 | 0.25, 0.70 |
| Intense or prolonged psychological distress at exposure to internal or external cues (B4) | 0.45 | 0.25, 0.65 |
| Markedly diminished interest or participation in significant activities (D5) | 0.43 | 0.23, 0.64 |
| Avoidance of or efforts to avoid external reminders (C2) | 0.39 | 0.22, 0.56 |
| Irritable behavior and angry outbursts (E1) | 0.37 | 0.10, 0.65 |
| Feelings of detachment or estrangement from others (D6) | 0.35 | 0.14, 0.56 |
| Sleep disturbance (E6) | 0.35 | 0.14, 0.56 |
| Recurrent distressing dreams with content and/or affect related to the traumatic event(s) (B2) | 0.35 | 0.17, 0.52 |
| Problems with concentration (E5) | 0.35 | 0.17, 0.52 |
| Recurrent, involuntary, and intrusive distressing memories of the traumatic event(s) (B1) | 0.32 | 0.14, 0.50 |
| Persistent and exaggerated negative beliefs or expectations (D2) | 0.32 | 0.14, 0.49 |
| Marked physiological reactions to internal or external cues (B5) | 0.31 | 0.13, 0.48 |
| Exaggerated startle response (E4) | 0.30 | 0.12, 0.47 |
| Conversion Disorder | 0.36 | 0.04, 0.67 |
| Symptoms of altered voluntary motor or sensory function (A) | 0.36 | 0.04, 0.67 |
| Agoraphobia | 0.35 | 0.17, 0.53 |
| Fear of using public transportation (A1) | 0.40 | 0.22, 0.57 |
| Fearing or avoiding situations (B) | 0.39 | 0.22, 0.56 |
| Fear of being outside of the home alone (A5) | 0.36 | 0.18, 0.54 |
| Fear of standing in line or being in a crowd (A4) | 0.31 | 0.14, 0.49 |
| Fear of being in open spaces (A2) | 0.30 | 0.12, 0.48 |
| Generalized Anxiety Disorder | 0.34 | 0.14, 0.54 |
| Restlessness or feeling keyed up or on edge (C1) | 0.50 | 0.29, 0.71 |
| Difficulty concentrating or mind going blank (C3) | 0.36 | 0.16, 0.56 |
| Sleep disturbance (C6) | 0.35 | 0.14, 0.56 |
| Being easily fatigued (C2) | 0.27 | 0.10, 0.45 |
| Irritability (C4) | 0.21 | -0.01, 0.44 |
| Panic Disorder | 0.26 | 0.08, 0.45 |
| Derealization or depersonalization (A11) | 0.39 | 0.12, 0.66 |
| Recurrent unexpected panic attacks (A) | 0.35 | 0.17, 0.52 |
| Sensations of shortness of breath or smothering (A4) | 0.35 | 0.17, 0.53 |
| Trembling or shaking (A3) | 0.29 | 0.12, 0.47 |
| Fear of losing control or 'going crazy' (A12) | 0.29 | 0.11, 0.47 |
| Chest pain or discomfort (A6) | 0.27 | 0.09, 0.44 |
| Chills or heat sensations (A9) | 0.22 | 0.04, 0.39 |
| Paresthesias (numbness or tingling sensations) (A10) | 0.20 | 0.02, 0.38 |
| Feeling dizzy, unsteady, light-headed, or faint (A8) | 0.18 | 0.00, 0.36 |
| Nausea or abdominal distress (A7) | 0.12 | -0.06, 0.30 |
| Obsessive-Compulsive Disorder | 0.25 | 0.06, 0.43 |
| Repetitive behaviors in response to an obsession or according to rules that must be applied rigidly (A2.1) | 0.25 | 0.06, 0.43 |
| Dissociative Amnesia | 0.10 | -0.18, 0.37 |
| Inability to recall important autobiographical information, usually of a traumatic or stressful nature (A) | 0.10 | -0.18, 0.37 |
| β: Standardized beta;P Pletters in parenthesis represent the DSM-5 criterion. | | |

| **Supplementary Table 14.** Functional Impairment Prediction Scale (FIPS) | | | | | | | | | | | | | | | | | |
| --- | --- | --- | --- | --- | --- | --- | --- | --- | --- | --- | --- | --- | --- | --- | --- | --- | --- |
| Instructions: How often do you have the following experiences when you are UnotU under the influence of alcohol or drugs? Please circle the number that best describes you. Circle a "0" if the experience never happens to you; circle a "10" if it is always happening to you. If it happens sometimes, but not all the time, circle a number between 1 and 9 that best describes how often it happens to you. | | | | | | | | | | | | | | | | | |
| Some people sometimes find that they are approached by people that they do not know who call them by another name or insist that they have met them before. | | | | | | | | | | | | | | | | | |
| 0%  (never) | 10 | 20 | | | 30 | 40 | | 50 | | 60 | 70 | | 80 | | 90 | | 100%  (always) |
| O | O | O | | | O | O | | O | | O | O | | O | | O | | O |
| Some people have the experience of being in a familiar place but finding it strange and unfamiliar. | | | | | | | | | | | | | | | | | |
| 0%  (never) | 10 | 20 | | | 30 | 40 | | 50 | | 60 | 70 | | 80 | | 90 | | 100%  (always) |
| O | O | O | | | O | O | | O | | O | O | | O | | O | | O |
| Some people find that when they are watching television or a movie they become so absorbed in the story that they are unaware of other events happening around them. | | | | | | | | | | | | | | | | | |
| 0%  (never) | 10 | 20 | | | 30 | 40 | | 50 | | 60 | 70 | | 80 | | 90 | | 100%  (always) |
| O | O | O | | | O | O | | O | | O | O | | O | | O | | O |
| Some people sometimes find that in certain situations they are able to do things with amazing ease and spontaneity that would usually be difficult for them (for example, sports, work, social situations, etc.). | | | | | | | | | | | | | | | | | |
| 0%  (never) | 10 | 20 | | | 30 | 40 | | 50 | | 60 | 70 | | 80 | | 90 | | 100%  (always) |
| O | O | O | | | O | O | | O | | O | O | | O | | O | | O |
| While watching TV, you find that you are thinking about something else. | | | | | | | | | | | | | | | | | |
| 0  (never) | 1 | 2 | | | 3 | 4 | | 5 | | 6 | 7 | | 8 | | 9 | | 10  (always) |
| O | O | O | | | O | O | | O | | O | O | | O | | O | | O |
| Feeling as if your body (or certain parts of it) are unreal. | | | | | | | | | | | | | | | | | |
| 0  (never) | 1 | 2 | | | 3 | 4 | | 5 | | 6 | 7 | | 8 | | 9 | | 10  (always) |
| O | O | O | | | O | O | | O | | O | O | | O | | O | | O |
| Having trance-like episodes where you stare off into space and lose awareness of what is going on around you. | | | | | | | | | | | | | | | | | |
| 0  (never) | 1 | 2 | | | 3 | 4 | | 5 | | 6 | 7 | | 8 | | 9 | | 10  (always) |
| O | O | O | | | O | O | | O | | O | O | | O | | O | | O |
| Thoughts being imposed on you or imposed on your mind. | | | | | | | | | | | | | | | | | |
| 0  (never) | 1 | 2 | | | 3 | 4 | | 5 | | 6 | 7 | | 8 | | 9 | | 10  (always) |
| O | O | O | | | O | O | | O | | O | O | | O | | O | | O |
| Being unable to remember your name, or age, or address. | | | | | | | | | | | | | | | | | |
| 0  (never) | 1 | 2 | | | 3 | 4 | | 5 | | 6 | 7 | | 8 | | 9 | | 10  (always) |
| O | O | O | | | O | O | | O | | O | O | | O | | O | | O |
| Being paralyzed or unable to move (for no known medical reason). | | | | | | | | | | | | | | | | | |
| 0  (never) | 1 | 2 | | | 3 | 4 | | 5 | | 6 | 7 | | 8 | | 9 | | 10  (always) |
| O | O | O | | | O | O | | O | | O | O | | O | | O | | O |
| Finding yourself lying in bed (on the sofa, etc.) with no memory of how you got there. | | | | | | | | | | | | | | | | | |
| 0  (never) | 1 | 2 | | | 3 | 4 | | 5 | | 6 | 7 | | 8 | | 9 | | 10  (always) |
| O | O | O | | | O | O | | O | | O | O | | O | | O | | O |
| Having difficulty walking (for no known medical reason). | | | | | | | | | | | | | | | | | |
| 0  (never) | 1 | 2 | | | 3 | 4 | | 5 | | 6 | 7 | | 8 | | 9 | | 10  (always) |
| O | O | O | | | O | O | | O | | O | O | | O | | O | | O |
| Hearing a lot of noise or yelling in your head. | | | | | | | | | | | | | | | | | |
| 0  (never) | 1 | 2 | | | 3 | 4 | | 5 | | 6 | 7 | | 8 | | 9 | | 10  (always) |
| O | O | O | | | O | O | | O | | O | O | | O | | O | | O |
| Re-experiencing body sensations from a past traumatic event. | | | | | | | | | | | | | | | | | |
| 0  (never) | 1 | 2 | | | 3 | 4 | | 5 | | 6 | 7 | | 8 | | 9 | | 10  (always) |
| O | O | O | | | O | O | | O | | O | O | | O | | O | | O |
| Feeling like you are 'inside' yourself, watching what you are doing. | | | | | | | | | | | | | | | | | |
| 0  (never) | 1 | 2 | | | 3 | 4 | | 5 | | 6 | 7 | | 8 | | 9 | | 10  (always) |
| O | O | O | | | O | O | | O | | O | O | | O | | O | | O |
| Feeling distant or removed from your thoughts and actions. | | | | | | | | | | | | | | | | | |
| 0  (never) | 1 | 2 | | | 3 | 4 | | 5 | | 6 | 7 | | 8 | | 9 | | 10  (always) |
| O | O | O | | | O | O | | O | | O | O | | O | | O | | O |
| Reliving a past trauma so vividly that you see it, hear it, feel it, smell it, etc. | | | | | | | | | | | | | | | | | |
| 0  (never) | 1 | 2 | | | 3 | 4 | | 5 | | 6 | 7 | | 8 | | 9 | | 10  (always) |
| O | O | O | | | O | O | | O | | O | O | | O | | O | | O |
| Your thoughts and feelings are so changeable that you don't understand yourself. | | | | | | | | | | | | | | | | | |
| 0  (never) | 1 | 2 | | | 3 | 4 | | 5 | | 6 | 7 | | 8 | | 9 | | 10  (always) |
| O | O | O | | | O | O | | O | | O | O | | O | | O | | O |
| Reliving a traumatic event so totally that you think that a present-day person is actually a person from the trauma (for example, being home with your partner, suddenly reliving being raped by your alcoholic uncle, and actually thinking that your partner is your uncle - that is, you see your uncle in front of you instead of seeing your partner). | | | | | | | | | | | | | | | | | |
| 0  (never) | 1 | 2 | | | 3 | 4 | | 5 | | 6 | 7 | | 8 | | 9 | | 10  (always) |
| O | O | O | | | O | O | | O | | O | O | | O | | O | | O |
| Feeling as if there is something inside you that takes control of your behavior or speech. | | | | | | | | | | | | | | | | | |
| 0  (never) | 1 | 2 | | | 3 | 4 | | 5 | | 6 | 7 | | 8 | | 9 | | 10  (always) |
| O | O | O | | | O | O | | O | | O | O | | O | | O | | O |
| Discovering that you have a significant injury (for example, a cut, or a burn, or many bruises), and having no memory of how it happened. | | | | | | | | | | | | | | | | | |
| 0  (never) | 1 | 2 | | | 3 | 4 | | 5 | | 6 | 7 | | 8 | | 9 | | 10  (always) |
| O | O | O | | | O | O | | O | | O | O | | O | | O | | O |
| Suddenly finding yourself somewhere (for example, at the beach, at work, in a nightclub, in your car, etc.) with no memory of how you got there. | | | | | | | | | | | | | | | | | |
| 0  (never) | 1 | 2 | | | 3 | 4 | | 5 | | 6 | 7 | | 8 | | 9 | | 10  (always) |
| O | O | O | | | O | O | | O | | O | O | | O | | O | | O |
| Some thoughts are suddenly 'taken away from you.' | | | | | | | | | | | | | | | | | |
| 0  (never) | 1 | 2 | | | 3 | 4 | | 5 | | 6 | 7 | | 8 | | 9 | | 10  (always) |
| O | O | O | | | O | O | | O | | O | O | | O | | O | | O |
| Feeling a struggle inside you about what to think, how to feel, what you should do. | | | | | | | | | | | | | | | | | |
| 0  (never) | 1 | 2 | | | 3 | 4 | | 5 | | 6 | 7 | | 8 | | 9 | | 10  (always) |
| O | O | O | | | O | O | | O | | O | O | | O | | O | | O |
| Instructions: These questions ask about different physical symptoms or body experiences, which you may have had either briefly or for a longer time. Please indicate to what extent these experiences apply to you Uin the past yearU. For each statement, please the number that best applies to you. | | | | | | | | | | | | | | | | | |
| I dislike smells that I usually like. | | | | | | | | | | | | | | | | | |
| not at all | | | a little | | | | moderately | | | | | quite a bit | | | | extremely | |
| O | | | O | | | | O | | | | | O | | | | O | |
| I cannot see for a while (as if I am blind). | | | | | | | | | | | | | | | | | |
| not at all | | | a little | | | | moderately | | | | | quite a bit | | | | extremely | |
| O | | | O | | | | O | | | | | O | | | | O | |
| I grow stiff for a while. | | | | | | | | | | | | | | | | | |
| not at all | | | a little | | | | moderately | | | | | quite a bit | | | | extremely | |
| O | | | O | | | | O | | | | | O | | | | O | |
| Instructions: Please circle the one statement that best describes the way you have been feeling during the Upast two weeks, including todayU. If several statements apply to equally well, circle the answer that is located towards right. | | | | | | | | | | | | | | | | | |
| I do not feel sad. | | | | I feel sad. | | | | | I am sad all the time and I can't snap out of it. | | | | | I am so sad and unhappy that I can't stand it. | | | |
| O | | | | O | | | | | O | | | | | O | | | |
| I make decisions about as well as I ever could. | | | | I put off making decisions more than I used to. | | | | | I have greater difficulty in making decisions more than I used to. | | | | | I can't make decisions at all anymore. | | | |
| O | | | | O | | | | | O | | | | | O | | | |
| I don't get more tired than usual. | | | | I get tired more easily than I used to. | | | | | I get tired from doing almost anything. | | | | | I am too tired to do anything. | | | |
| O | | | | O | | | | | O | | | | | O | | | |
| My appetite is no worse than usual. | | | | My appetite is not as good as it used to be. | | | | | My appetite is much worse now. | | | | | I have no appetite at all anymore. | | | |
| O | | | | O | | | | | O | | | | | O | | | |
| I am no more worried about my health than usual. | | | | I am worried about physical problems like aches, pains, upset stomach, or constipation. | | | | | I am very worried about physical problems and it's hard to think of much else. | | | | | I am so worried about my physical problems that I cannot think of anything else. | | | |
| O | | | | O | | | | | O | | | | | O | | | |
| I have not noticed any recent change in my interest in sex. | | | | I am less interested in sex than I used to be. | | | | | I have almost no interest in sex. | | | | | I have lost interest in sex completely. | | | |
| O | | | | O | | | | | O | | | | | O | | | |
| Instructions: Please indicate how much that problem has distressed or bothered you during the Upast 7 days including today | | | | | | | | | | | | | | | | | |
| Feeling afraid in open spaces. | | | | | | | | | | | | | | | | | |
| not at all | | | a little bit | | | | moderately | | | | | quite a bit | | | | extremely | |
| O | | | O | | | | O | | | | | O | | | | O | |
| Thoughts of ending your life. | | | | | | | | | | | | | | | | | |
| not at all | | | a little bit | | | | moderately | | | | | quite a bit | | | | extremely | |
| O | | | O | | | | O | | | | | O | | | | O | |
| Feeling blocked in getting things done. | | | | | | | | | | | | | | | | | |
| not at all | | | a little bit | | | | moderately | | | | | quite a bit | | | | extremely | |
| O | | | O | | | | O | | | | | O | | | | O | |
| Feeling blue. | | | | | | | | | | | | | | | | | |
| not at all | | | a little bit | | | | moderately | | | | | quite a bit | | | | extremely | |
| O | | | O | | | | O | | | | | O | | | | O | |
| Feeling no interest in things. | | | | | | | | | | | | | | | | | |
| not at all | | | a little bit | | | | moderately | | | | | quite a bit | | | | extremely | |
| O | | | O | | | | O | | | | | O | | | | O | |
| Difficulty making decisions. | | | | | | | | | | | | | | | | | |
| not at all | | | a little bit | | | | moderately | | | | | quite a bit | | | | extremely | |
| O | | | O | | | | O | | | | | O | | | | O | |
| Feeling afraid to travel on buses, subways, or trains. | | | | | | | | | | | | | | | | | |
| not at all | | | a little bit | | | | Moderately | | | | | quite a bit | | | | extremely | |
| O | | | O | | | | O | | | | | O | | | | O | |
| Trouble getting your breath. | | | | | | | | | | | | | | | | | |
| not at all | | | a little bit | | | | moderately | | | | | quite a bit | | | | extremely | |
| O | | | O | | | | O | | | | | O | | | | O | |
| Feeling weak in parts of your body. | | | | | | | | | | | | | | | | | |
| not at all | | | a little bit | | | | moderately | | | | | quite a bit | | | | extremely | |
| O | | | O | | | | O | | | | | O | | | | O | |
| Feeling tense or keyed up. | | | | | | | | | | | | | | | | | |
| not at all | | | a little bit | | | | moderately | | | | | quite a bit | | | | extremely | |
| O | | | O | | | | O | | | | | O | | | | O | |
| Feeling very self-conscious with others. | | | | | | | | | | | | | | | | | |
| not at all | | | a little bit | | | | moderately | | | | | quite a bit | | | | extremely | |
| O | | | O | | | | O | | | | | O | | | | O | |
| Never feeling close to another person. | | | | | | | | | | | | | | | | | |
| not at all | | | a little bit | | | | moderately | | | | | quite a bit | | | | extremely | |
| O | | | O | | | | O | | | | | O | | | | O | |
| Spells of terror or panic. | | | | | | | | | | | | | | | | | |
| not at all | | | a little bit | | | | moderately | | | | | quite a bit | | | | extremely | |
| O | | | O | | | | O | | | | | O | | | | O | |
| Feeling nervous when you are left alone. | | | | | | | | | | | | | | | | | |
| not at all | | | a little bit | | | | moderately | | | | | quite a bit | | | | extremely | |
| O | | | O | | | | O | | | | | O | | | | O | |
